# Cancer Stem Cells Shift Metabolite Acetyl‐Coenzyme A to Abrogate the Differentiation of CD103^+^ T Cells

**DOI:** 10.1002/advs.202513535

**Published:** 2025-11-23

**Authors:** Jiaxin Lei, Huiyan Ji, Jing Guo, Mengdi Liu, Danhua Su, Yiran Zheng, Lin Xu, Qinghua Cao, Tao Ren, Jun Gui, Zhenke Wen

**Affiliations:** ^1^ The Fourth Affiliated Hospital of Soochow University Institutes of Biology and Medical Sciences Suzhou Medical College of Soochow University Suzhou 215000 China; ^2^ Jiangsu Key Laboratory of Infection and Immunity MOE Key Laboratory of Geriatric Diseases and Immunology Soochow University Suzhou 215000 China; ^3^ College of Pharmaceutical Sciences Suzhou Medical College of Soochow University Suzhou 215000 China; ^4^ Department of Immunology Zunyi Medical University Zunyi 563000 China; ^5^ Renal Medicine Kolling Institute of Medical Research Sydney Medical School The University of Sydney St Leonards NSW 2065 Australia; ^6^ Royal North Shore Hospital St Leonards NSW 2065 Australia; ^7^ Department of Respiratory Medicine Shanghai Jiao Tong University Affiliated Sixth People's Hospital Shanghai 200233 China; ^8^ State Key Laboratory of Systems Medicine for Cancer Ren Ji Hospital Shanghai Jiao Tong University School of Medicine Shanghai 200127 China

**Keywords:** acetyl‐CoA, cancer stem cell, CD103+ T cell

## Abstract

CD103^+^ T cells mediate potent anti‐tumor immune responses and correlate with favorable clinical outcomes in cancer patients. However, the mechanisms by which cancer cells influence the differentiation of these cells remain elusive. Herein, we demonstrate that cancer stem cells (CSCs) play a pivotal role in suppressing CD103^+^ T cell differentiation in patients with non‐small cell lung cancer (NSCLC). Specifically, CSCs facilitate the transfer of the metabolite acetyl‐coenzyme A (acetyl‐CoA) into interacting T cells via an exosome‐dependent pathway. This process enhances the acetylation of B lymphocyte‐induced maturation protein 1 (Blimp‐1), a critical transcription factor governing CD103^+^ T cell differentiation. Acetylation of Blimp‐1 strengthens its interaction with the E3 ubiquitin ligase LIS1, thereby promoting Blimp‐1 degradation, which ultimately blocks CD103^+^ T cell differentiation. Accordingly, targeting CSCs and acetyl‐CoA biosynthesis using CD133 antibody‐conjugated nanoparticles increases tumor‐infiltrating CD8^+^CD103^+^ T cells and suppresses tumor growth. Importantly, studies using NSCLC patient‐derived organoids (PDOs) and humanized PDO‐NSG chimeras confirmed that blocking acetyl‐CoA production, exosome secretion from CSCs, and key enzymes involved in Blimp‐1 acetylation and ubiquitination effectively restores CD103^+^ T cell differentiation. Altogether, CSC acetyl‐CoA is a key contributor in impairing CD103^+^ T cells through programming post‐translational modifications, serving as a promising therapeutic target in anti‐tumor therapy.

## Graphical abstract

Lei et al. demonstrate that cancer stem cells (CSCs) play a pivotal role in impairing the differentiation of CD103+ T cells in patients with non‐small‐cell lung cancer. The key mechanism involves CSC‐derived acetyl‐CoA, which disrupts CD103+ T cell differentiation by sequentially inducing acetylation and ubiquitination of the Blimp‐1 protein. Targeting this pathway offers a promising therapeutic strategy for anti‐tumor therapy.

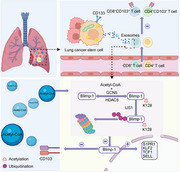

## 1 Introduction

Lung cancer remains the leading cause of cancer‐related death, with non‐small‐cell lung cancer (NSCLC) as the dominant subtype accounting for ≈85% of clinical patients.^[^ ^1^, ^2^ ^]^ For patients with unresectable disease, immunotherapy integrated with chemoradiation is becoming the new standard treatment.^[^ ^3^ ^]^ The development and progression of cancer are closely related to the interactions between cancer cells and anti‐tumor T cells within the tumor microenvironment (TME). The TME represents a complex ecosystem comprising fibroblasts, endothelial cells, and both innate and adaptive immune cells.^[^ ^4^ ^]^ Tumor cells can dysregulate immune checkpoint protein expression on T cells, constituting a key immune evasion mechanism.^[^ ^5^ ^]^ This rationale supports the therapeutic application of immune checkpoint blockade (ICB) in the TME.^[^ ^6^, ^7^ ^]^ However, the majority of patients do not respond to regimens including ICB in clinic practice, which can even result in a series of organ‐specific inflammation, even multiorgan failure.^[^ ^8^, ^9^ ^]^ Accordingly, mechanistic investigations exploring the deficiency and dysfunction of anti‐tumor T cells are relevant for optimizing clinical treatments.

In recent years, a subpopulation of tumor‐reactive CD8^+^CD103^+^ T cells was identified in TME. Different from central memory (Tcm) and effector memory (Tem) subsets that resident in lymphoid organs or circulate in blood, CD103^+^ T cells usually persist in peripheral tissues and do not recirculate.^[^ ^10^, ^11^ ^]^ Specifically, such cells frequently co‐express CD69, a C‐type lectin that antagonizes S1PR1, which mediates T cell egress, enforcing a tissue‐resident state.^[^ ^12^ ^]^ While CCR7^+^ T cells continue to migrate into the afferent lymphoid tissue and accumulate in lymph nodes, CD103^+^ T cells express low levels of CCR7. Meanwhile, CD103^+^ T cells may express CD49a, which constitutes the α‐subunit of the α1β1 integrin receptor for binding to extracellular matrix such as collagen.^[^ ^13^ ^]^ Some studies suggest that CD103^+^ T cells originate from effector T cells and differentiate into memory cells through integrated signaling involving transcription factors, cell surface molecules, and cytokines. Key transcriptional regulators of CD103^+^ T cell programming include B lymphocyte‐induced maturation protein 1 (Blimp‐1), homolog of Blimp‐1 in T cells (Hobit), Notch, Eomesodermin (Eomes), runt‐related transcription factor 1 (RUNX1), and runt‐related transcription factor 3 (RUNX3).^[^ ^14^, ^15^ ^]^ However, the molecular mechanisms governing CD103^+^ T cell differentiation in TME remain poorly defined.

CD103^+^ T cells persist in peripheral tissues, providing fast and efficient responses to reinfection.^[^ ^11^ ^]^ In addition to being present at pathogen entry sites such as mucous membranes, CD103^+^ T cells play a role in internal organs, including the liver and brain,^[^ ^16^ ^]^ striking a balance between infection control and immunopathological damage. It is reported that CD103^+^ T cells can respond to autoantigens, leading to autoimmune disorders such as autoimmune systemic lupus erythematosus and rheumatoid arthritis.^[^ ^17^, ^18^ ^]^ In patients with lung cancer and many other solid tumors, CD103^+^ T cell infiltration is associated with improved prognosis.^[^ ^19^ ^]^ CD103‐expressing cytotoxic CD8^+^ T cells exhibit enhanced antitumor activity.^[^ ^20^ ^]^ In NSCLC specimens, CD8^+^CD103^+^ T cells exhibit robust secretion of IFN‐γ and granzyme B, along with elevated lysosomal‐associated membrane protein 1 (LAMP1 /CD107a) expression, collectively mediating potent tumor cell killing.^[^ ^19^, ^21^ ^]^ These findings suggest that restoring the abundance and function of CD103^+^ T cells in the TME may represent a novel therapeutic strategy for cancer immunotherapy.

It is well‐known that cancer cells have substantial intra‐tumor heterogeneity, consisting of cancer stem cells (CSCs) and non‐CSCs. Several markers, such as CD133, CD44, CD166, CD24, and ALDH1, have proven useful for prospective isolation of CSCs in multiple solid tumors.^[^ ^22^ ^]^ Importantly, CSCs play a critical role in tumor metastasis and drug resistance. Since current anti‐cancer therapies primarily target non‐CSCs, they often fail to eradicate CSCs and may even promote the expansion of the CSC pool. This leads to treatment resistance and subsequent relapse in cancer patients.^[^ ^22^ ^]^ Meanwhile, accumulating evidence suggests that CSCs are distinct from non‐CSC tumor cells not only phenotypically and functionally but also metabolically. While an increased glycolytic metabolism is well acknowledged as the metabolic characteristic for non‐CSC tumor cells, several studies have implicated an essential role of mitochondrial oxidative phosphorylation for CSC maintenance.^[^ ^23^ ^]^ In consistent, our recent studies have identified that elevated mitochondrial fusion rates are critical for maintaining mitochondrial quality and promoting NSCLC CSC expansion. Specifically, NSCLC CSCs exert robust lipogenesis to drive optic atrophy 1 (OPA1) transcription and enhance mitochondrial fusion. Consequently, targeting lipogenesis and mitochondrial fusion effectively inhibits the tumor outgrowth.^[^ ^24^ ^]^ While these findings clearly demonstrate the metabolic adaptations of CSCs, whether and how CSCs modulate CD103^+^ T cell differentiation remains largely unknown.

In this study, we investigated the differentiation of CD103^+^ T cells in the TME of NSCLC by characterizing the effects of CSCs and non‐CSCs separately. We identified a critical role for CSCs in inhibiting CD103^+^ T cell differentiation. Mechanistically, NSCLC CSCs were found to transfer the lipogenic carbon source acetyl‐CoA to interacting T cells via exosomes, inducing acetylation and subsequent ubiquitination of the Blimp‐1 protein, thereby suppressing CD103^+^ T cell differentiation. These findings reveal a previously unknown function of CSCs in the TME and suggest promising therapeutic targets for improving clinical management of NSCLC.

## 2 Results

### 2.1 CSCs are Functional and Essential for Inhibiting the Differentiation of CD103^+^ T Cells

**Figure 1:**
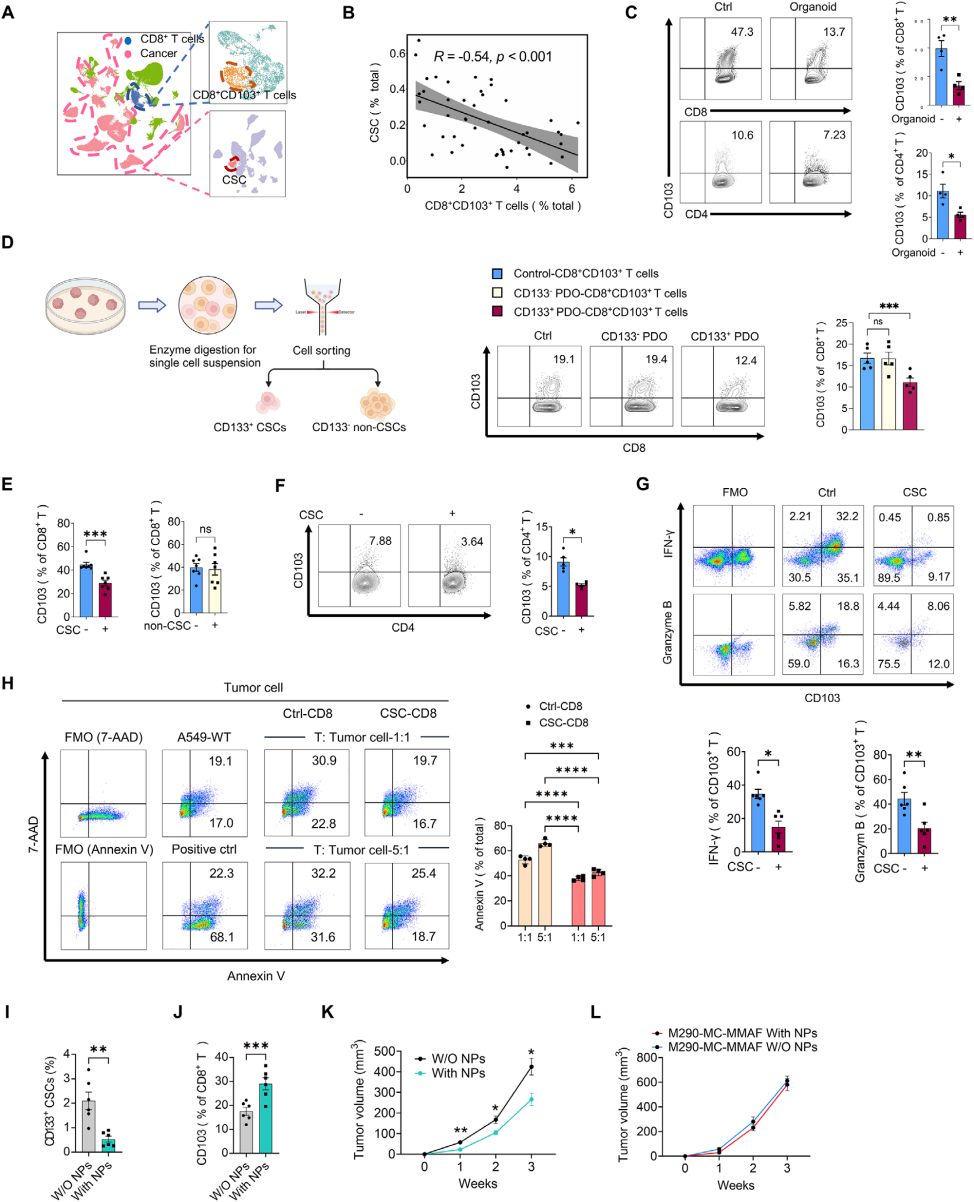
Functional CSCs impede the differentiation of CD103^+^ T cells. A) UMAP plot of NSCSL patients, colored by CD8^+^CD103^+^ T cells, CSCs, and other cells. B) The correlation between the numbers of CD133^+^ CSCs and CD8^+^CD103^+^ T cells in NSCLC patients. C) CD8^+^ T cells and CD4^+^ T cells isolated from PBMCs of healthy donors were pre‐conditioned with or without PDOs for 24 h, followed by induction of CD103^+^ T cells with anti‐CD3/CD28 beads plus TGF‐β (10 ng mL^−1^) for 72 h. The percentage of CD8^+^CD103^+^ and CD4^+^CD103^+^ T cells was assessed by flow cytometry. Mean ± SEM from 4 individuals. D) Healthy CD8^+^ T cells were co‐cultured with PDOs‐derived primary CD133^−^ or CD133^+^ tumor cells for 24 h, followed by induction of CD103^+^ T cells ex vivo. Mean ± SEM from 5 individuals. E) Healthy CD8^+^ T cells were pre‐conditioned with CSCs or non‐CSCs cultured from A549 cells for 24 h, then activated with anti‐CD3/CD28 beads plus TGF‐β (10 ng mL^−1^) for 72 h. The percentage of CD8^+^CD103^+^ T cells was assessed by flow cytometry. Data are presented as mean ± SEM from 7 individuals per group. F) Healthy CD4^+^ T cells were co‐cultured with or without CSCs for 24 h, followed by induction of CD103^+^ T cells ex vivo. Mean ± SEM from 5 individuals in each group. G) Healthy CD8^+^ T cells were co‐cultured with or without CSCs for 24 h, followed by induction of CD103^+^ T cells ex vivo. The percentages of CD8^+^CD103^+^IFN‐γ^+^ and CD8^+^CD103^+^Granzyme B^+^ T cells were measured by flow cytometry. Data are mean ± SEM from 6 individuals. H) Percentage of Annexin‐V^+^ A549 after incubation with activated CSC‐conditioned CD8^+^ T cells or control CD8^+^ T cells for 96 h. Mean ± SEM from 4 individuals. I–K) Syngeneic LLC mice were treated by intra‐tumor injection of CD133 antibody‐conjugated NPs carrying DAPT, followed by detecting Pan‐CK^+^CD133^+^ CSCs, CD8^+^CD103^+^ T cells, and tumor growth. Mean ± SEM from 6 mice in each group. L) Syngeneic LLC‐bearing mice were pretreated with the M290‐MC‐MMAF, followed by treatment with CD133 antibody‐conjugated NPs carrying DAPT. Mean ± SEM from 6 mice in each group. *p < 0.05, **p < 0.01, ***p < 0.001 and ****p < 0.0001 with paired *t*‐test C, E–G, I.J), ANOVA plus Tukey's method D, H, K,L) and Pearson correlation statistical analysis (B).

To explore the possible correlations between CSCs and CD103^+^ T cells in human NSCLC, we analyzed the expression of CD133, the classical marker for CSCs, and CD103 using the Gene Expression Omnibus (GEO) database of NSCLC tissues from clinical patients. Of interest, we observed that the expression of CD133 in CSCs was negatively associated with the expression of CD103 in CD8^+^ T cells in NSCLC tissues (**Figure** **1**A,B). Given the observed spatial proximity of these cell populations in the TME (Figure S1, Supporting Information), we hypothesized that CSCs may locally suppress CD103⁺ T cell differentiation.

As transforming growth factor‐β (TGF‐β) plays a critical role in the development of CD103^+^ T cells through the TGFβ‐SMAD3‐dependent pathway,^[^ ^25^ ^]^ ex vivo differentiation of those T cells by activating T cells in the presence of TGF‐β has been well established.^[^ ^26^, ^27^ ^]^ We confirmed that activation of CD8^+^ T cells with anti‐CD3/CD28 beads plus TGF‐β was efficient in driving CD103^+^ T cells (Figure S2A–C, Supporting Information). To overcome the limited access to patient‐derived tumor cells, NSCLC PDOs were established as evidenced by H&E staining with immunostainings of PanCK plus CD31 (Figure S3A–C, Supporting Information). We consistently found that NSCLC PDOs efficiently inhibited the CD103^+^ T cell differentiation of healthy CD8^+^ and CD4^+^ T cells (Figure 1C; Figure S4A, Supporting Information). After confirming that CD133⁺ tumor cells exhibit significantly enhanced stem‐like properties compared to CD133^−^ tumor cells (Figure S4B–E, Supporting Information), we cocultured primary tumor cells with CD8^+^ T cells. Notably, CD133^−^ tumor cells isolated from these PDOs had no significant effect on CD103⁺ T cell differentiation (Figure 1D), while CD133^+^ primary CSCs efficiently impeded the CD103^+^ T cell differentiation (Figure 1D), assigning an essential function to CSCs in blocking CD103^+^ T cell differentiation.

To reassure the inhibitory effect of CSCs on CD103^+^ T cell differentiation, we enriched both CSCs and non‐CSCs ex vivo (Figure S4F, Supporting Information), followed by co‐culture with healthy CD8^+^ T cells and subsequent induction of CD103^+^ T cells by anti‐CD3/CD28 beads plus TGF‐β.^[^ ^26^ ^]^ Pre‐incubation of healthy CD8^+^ T cells with CSCs (ratio 1:1) but not non‐CSCs (ratio 1:1) could abrogate the differentiation of CD103^+^ T cells, as assessed by both cell frequency (Figure 1E) and signature gene expression (Figure S4G, Supporting Information). Such phenomena were also confirmed with an increased ratio of non‐CSCs to T cells (5:1) and a decreased ratio of CSCs to T cells (1:5), showing impaired differentiation of CD8^+^CD103^+^ T cells in response to CSCs (Figure S4H, Supporting Information). Of note, we found that neither CSCs nor non‐CSCs exerted significant effects on T cell activation and proliferation (Figure S4I,J, Supporting Information), ruling out their differential involvement in these processes. CD8^+^CD127^low^KLRG‐1^high^ effector T cells were insusceptible to CSCs (Figure S4K, Supporting Information). CSCs suppressed Th1/Th2/Th17 differentiation and promoted Treg generation—effects similar to those of non‐CSCs (Figure S4L, Supporting Information).^[^ ^28^ ^]^ Notably, CSCs showed no detectable impact on other memory T cell subsets (Figure S4M,N, Supporting Information) and the T cell exhaustion markers after 72 h stimulation (Figure S4O, Supporting Information), suggesting that CD103^+^ T cells may represent a selectively regulated population. In addition, compared to non‐CSCs, CSCs inhibited the maturation of DCs and promoted M2 macrophage polarization (Figure S4P, Supporting Information).

The inhibitory effect of CSCs on CD103^+^ T cells extended beyond CD8^+^ T cells, as pre‐incubation with healthy CD4^+^ T cells similarly impaired CD4^+^CD103^+^ T cell differentiation (Figure 1F), consistent with our observations in PDOs (Figure 1C). Notably, we found that CSCs not only suppressed CD103^+^ T cell differentiation but also diminished the production of anti‐tumor cytokines, including IFN‐γ and Granzyme B (Figure 1G). Correspondingly, CSC‐pretreated CD8^+^ T cells exhibited reduced LAMP1 (CD107a) expression (Figure S5A, Supporting Information), and CSCs consistently attenuated CD8^+^ T cell cytotoxic activity (Figure 1H). These findings were further validated in H1299 and Calu‐1 NSCLC cell lines, which demonstrated CSC‐mediated suppression of CD103^+^ T cell differentiation (Figure S5B,C, Supporting Information).

To test the effect of CSCs in the differentiation of CD103^+^ T cells in vivo under physiological conditions with a fully functional immune system, a syngeneic Lewis lung carcinoma (LLC) model was established in C57BL/6 mice, followed by intra‐tumor injection of CD133 antibody‐conjugated nanoparticles (NPs) carrying gamma‐secretase inhibitor DAPT, which is known to impede CSCs by blocking the Notch activity. Such nanoparticles selectively targeted CD133‐expressing CSCs (Figure S5D, Supporting Information) without observable off‐target accumulation (Figure S5E, Supporting Information). This treatment resulted in a decreased frequency of CSCs, an elevated frequency of CD8^+^CD103^+^ T cells, and impaired tumor growth in those syngeneic model mice (Figure 1I–K; Figure S5F–H, Supporting Information). Of note, the antitumor effect of those DAPT‐NPs was mediated by CD103^+^ T cells, given that eliminating these cells nullified their effect (Figure 1L). Together, NSCLC CSCs are critical players in blocking the differentiation of CD103^+^ T cells.

### 2.2 CSCs‐Mediated Blimp‐1 Ubiquitination Attenuates CD103^+^ T Cell Differentiation

**Figure 2:**
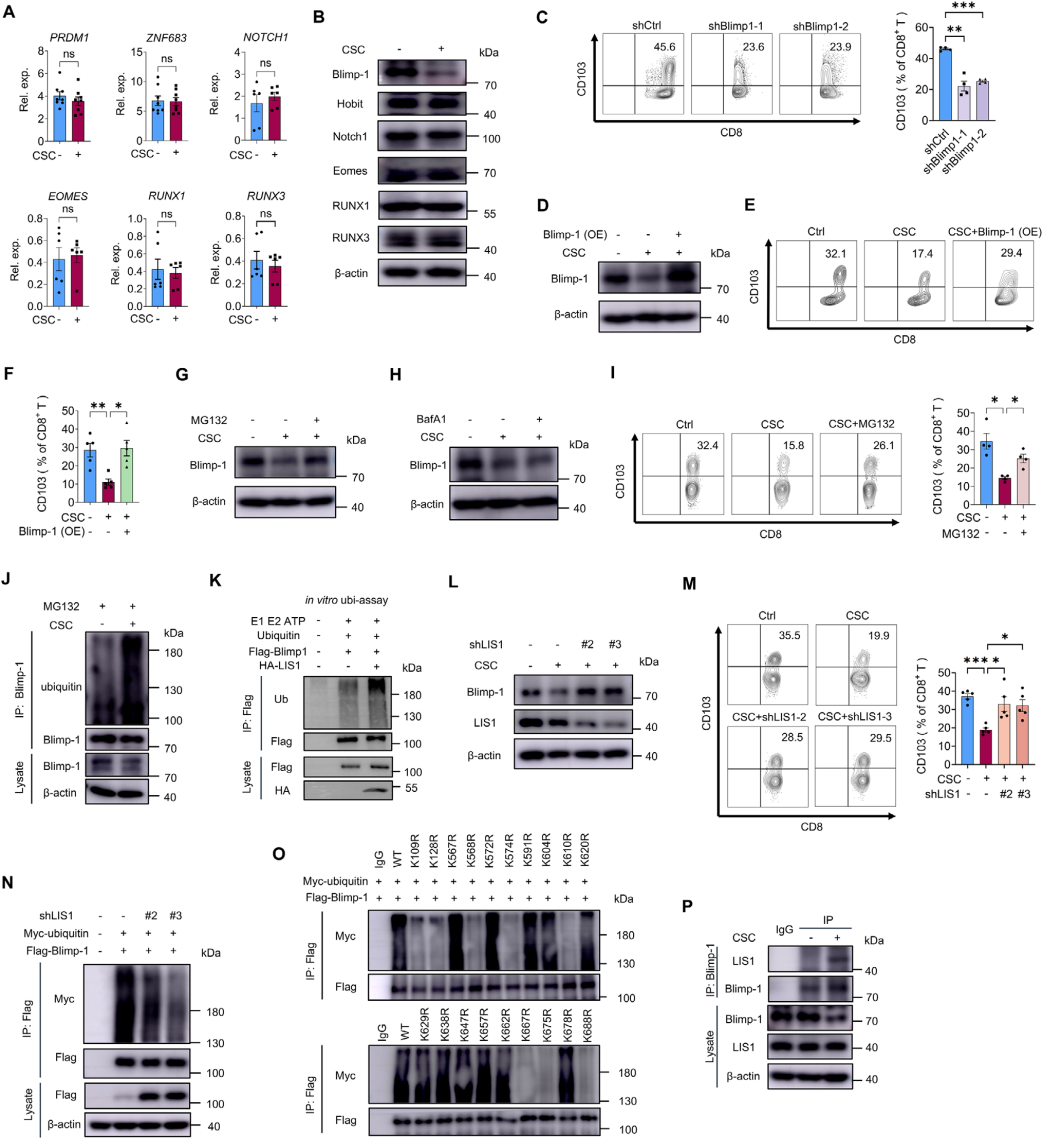
CSCs facilitate the ubiquitination of Blimp‐1 protein. A,B) CD8⁺ T cells were co‐cultured with or without CSCs for 24 h, followed by ex vivo induction of CD8⁺CD103⁺ T cells. mRNA and protein levels of the indicated transcription factors (Blimp‐1, Hobit, Notch1, Eomes, RUNX1, and RUNX3) were assessed by qPCR and immunoblotting. Mean ± SEM from 6 to 8 individuals in each group. C) CD8^+^CD103^+^ T cells were induced ex vivo after transfected with specific shRNAs against Blimp‐1. The percentage of CD8^+^CD103^+^ T cells was assessed by flow cytometry. Mean ± SEM from 4 individuals in each group. D–F) CSC‐conditioned CD8^+^ T cells were transfected with a Blimp‐1 plasmid via electroporation, followed by ex vivo induction of CD103^+^ T cells. Blimp‐1 protein expression and CD8^+^CD103^+^ T cell percentages were analyzed by immunoblotting and flow cytometry. Mean ± SEM from 5 individuals in each group. G–I) CD8⁺ T cells were educated with or without CSCs and then induced to differentiate into CD8⁺CD103⁺ T cells ex vivo. During induction, cells were treated with the proteasome inhibitor MG132 (2 µm), the lysosome inhibitor bafilomycin A1 (0.1 µm), or vehicle control for 4 h. Blimp‐1 expression was assessed by immunoblotting, and CD8^+^CD103⁺ T cell differentiation was evaluated by flow cytometry. Mean ± SEM from 4 individuals in each group. J) Endogenous Blimp‐1 was immunoprecipitated from induced T cells to analyze its ubiquitination status with or without CSC pretreatment. K) In vitro ubiquitination assay for analysis of Flag‐Blimp‐1 ubiquitination induced by purified LIS1 proteins. Flag‐Blimp‐1 was immunoprecipitated and subjected to Western blot. L,M) Immunoblot analysis of Blimp‐1 protein and flow cytometry analysis of CD8^+^CD103^+^ T cells were performed in CSC‐conditioned CD8^+^ T cells transfected with or without independent LIS1 shRNAs. N) Immunoprecipitation analysis of ubiquitination of Blimp‐1 in HEK293T cells that were co‐transfected with Flag‐Blimp‐1 and LIS1 shRNAs. O) Immunoprecipitation analysis of Blimp‐1 ubiquitination in HEK293T cells that were co‐transfected with Myc‐Ub and Flag‐Blimp‐1 mutants. P) Co‐immunoprecipitation assay assessing the interaction between endogenous Blimp‐1 and LIS1 in CD8⁺CD103⁺ T cells, with or without CSC pretreatment. *p < 0.05, **p < 0.01, and ***p < 0.001 with ANOVA plus Tukey's method (C, F, I, M).

Preincubation of CSCs with T cells did not affect the TGF‐β‐Smad3 signaling in T cells (Figure S6, Supporting Information), assigning a TGF‐β‐independent and T cell‐intrinsic mechanism underpinning impaired CD103^+^ T cell differentiation. Thus, we analyzed the expressions of transcription factors, including Blimp‐1 (encoded by *PRDM1*), Hobit (encoded by *ZNF683*), Notch1, Eomes, RUNX1, and RUNX3. Our analysis revealed a selective and sustained downregulation of Blimp‐1 protein—without changes in mRNA levels—following CSC pretreatment (**Figure** **2**A,B; Figure S7A, Supporting Information). Importantly, Blimp‐1 knockdown impaired CD103^+^ T cell differentiation (Figure 2C; Figure S7B, Supporting Information), while enforced Blimp‐1 expression rescued the CSC‐mediated suppression of differentiation, increasing both Blimp‐1 protein levels and the frequency of CD8^+^CD103^+^ T cells (Figure 2D–F). These findings establish Blimp‐1 deficiency as essential for CSC‐induced inhibition of CD103^+^ T cell differentiation.

Given that Blimp‐1 was downregulated at the protein but not mRNA level, we investigated whether the ubiquitin‐proteasome pathway mediated Blimp‐1 deficiency in T cells. Treatment with the proteasome inhibitor MG132 restored Blimp‐1 protein levels in CSC‐pretreated T cells (Figure 2G), whereas inhibition of lysosomal degradation with BafA1 showed no significant effect (Figure 2H). To further confirm that CSC accelerates Blimp‐1 degradation rather than affecting its synthesis, CHX chase experiments demonstrated that CSC‐coculture significantly reduces the half‐life of Blimp‐1 (Figure S7C, Supporting Information). Moreover, MG132 treatment rescued the CD103^+^ T cell differentiation (Figure 2I). Supporting these findings, immunoprecipitation assays demonstrated increased Blimp‐1 ubiquitination in T cells after CSC preincubation (Figure 2J).

To characterize Blimp‐1 ubiquitination, we predicted 13 potential E3 ligases for Blimp‐1 using the UbiBrowser database (Figure S8A, Supporting Information), with LIS1 emerging as the most enriched candidate in T cells (Figure S8B, Supporting Information). We confirmed a physical interaction between LIS1 and immunoprecipitated Blimp‐1 in T cells (Figure S8C, Supporting Information). Additionally, an in vitro ubiquitination assay provided direct biochemical evidence that LIS1 possesses intrinsic E3 ubiquitin ligase activity toward Blimp‐1 (Figure 2K). Subsequently, we employed two independent shRNAs to knock down LIS1 expression (Figure S8D, Supporting Information) and found that LIS1 depletion consistently prevented both Blimp‐1 protein degradation and CSC‐induced impairment of CD103^+^ T cell differentiation (Figure 2L,M). In support, LIS1 knockdown efficiently reduced the ubiquitination of immunoprecipitated Blimp‐1 protein (Figure 2N). Importantly, LIS1 depletion also restored LAMP1 expression (Figure S8E, Supporting Information), further supporting the functional recovery. Such findings assign LIS1 as the critical E3 ligase to Blimp‐1 in CSC‐T cell interaction. Furthermore, we compared the conservative lysine residues in the Blimp‐1 amino acid sequence and mutated each lysine residue to arginine in sequence (Figure S8F, Supporting Information), and identified multiple lysine residues, including K109, K128, K568, K574, K610, K667, K675, and K688 for Blimp‐1 ubiquitination (Figure 2O). These results suggest that CSCs drive the ubiquitination and degradation of Blimp‐1 protein to inhibit CD103^+^ T cell differentiation.

CSCs did not affect the mRNA and protein levels of LIS1 in T cells (Figure S8G,H, Supporting Information). However, CSCs preincubation enhanced the physical binding of LIS1 protein to immunoprecipitated Blimp‐1 protein (Figure 2P), indicating that CSCs might shape post‐translational modifications (PTMs) of Blimp‐1 to modulate its protein interactions.

### 2.3 CSCs‐Derived Acetyl‐CoA Triggers the Degradation of Blimp‐1 in T Cells

**Figure 3:**
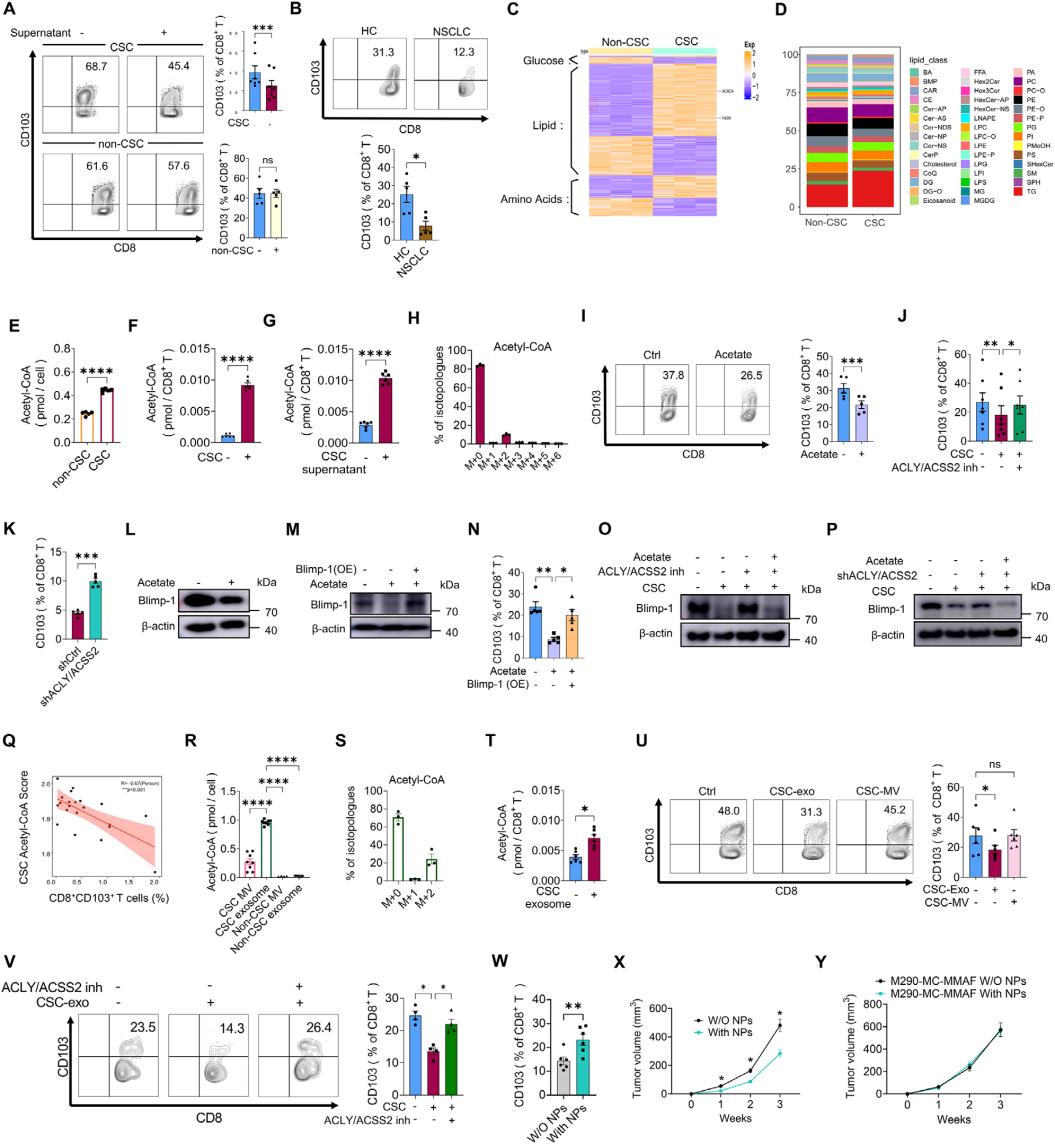
Acetyl‐CoA from CSCs licenses Blimp‐1 degradation. A) CD8^+^ T cells were pre‐incubated with CSCs or non‐CSCs‐conditioned supernatant for 24 h, and then induced to CD8^+^CD103^+^ T cells ex vivo. Mean ± SEM from 5 to 7 individuals in each group. B) CD8^+^ T cells from NSCLC patients and healthy controls were isolated, followed by induction of CD103^+^ T cells. Mean ± SEM from 5 individuals in each group. C) Heatmap showed metabolic signatures by quantifying the expression of glucose‐, lipid‐, and amino acid metabolism‐related genes that were differentially expressed in CSCs compared with non‐CSCs. RNA‐Seq from 3 samples in each group. D) Metabolomic analysis showed increased lipid content in CSCs. Metabolomic data from 3 samples in each group. E) Acetyl‐CoA levels in CSCs and non‐CSCs are shown. Data represent mean ± SEM from 5 independent experiments. F,G) Acetyl‐CoA levels in CD8^+^ T cells treated with CSCs or CSC‐conditioned supernatant for 24 h. Each dot represents one individual. H) After co‐culture of ^13^C‐acetate pre‐labeled CSCs with primary CD8⁺ T cells, the T cells were isolated and analyzed by UPLC‐MS for intracellular ^13^C‐labeled acetyl‐CoA. I) CD8^+^ T cells were induced to CD8^+^CD103^+^ T cells ex vivo in the presence or absence of acetate (10 mm), and the CD8^+^CD103^+^ T cell populations were analyzed by flow cytometry. Mean ± SEM from 5 individuals in each group. J) CSCs were pretreated with the ACLY inhibitor BMS‐303141 (40 µm) and the ACSS2 inhibitor (10 µm) for 24 h, followed by coculture with healthy CD8^+^ T cells for an additional 24 h. CD8^+^CD103^+^ T cells were induced ex vivo, and their percentage was quantified by flow cytometry. Mean ± SEM from 7 individuals in each group. K) CSCs were transfected with shRNAs targeting both ACLY and ACSS2 prior to conditioning healthy CD8^+^ T cells. CD8^+^CD103^+^ T cells were subsequently induced and quantified. Mean ± SEM from 5 individuals in each group. L) Blimp‐1 protein levels in induced T cells treated with or without acetate (10 mm) were assessed by immunoblotting. M,N) Ex vivo induction of CD8^+^CD103^+^ T cells with acetate (10 mm) ± Blimp‐1 overexpression. Blimp‐1 protein expression and CD8^+^CD103^+^ T cell differentiation were analyzed by immunoblotting and flow cytometry. Mean ± SEM from 5 individuals in each group. O) CD8^+^ T cells were cocultured with CSCs pretreated ± (BMS‐303141 and ACSS2 inhibitor) for 24 h, followed by ex vivo CD8^+^CD103^+^ induction ± acetate (10 mm). Blimp‐1 was analyzed by immunoblotting. P) CSCs transfected with ACLY and ACSS2‐targeting shRNAs conditioned CD8^+^ T cells, followed by ex vivo CD8^+^CD103^+^ induction ± acetate (10 mm). Blimp‐1 was assessed by immunoblotting. Q) Correlation between CD8^+^CD103^+^ T cell abundance and CSC acetyl‐CoA scores in NSCLC patients. R) Acetyl‐CoA levels in MVs and exosomes isolated from CSCs versus non‐CSCs. Mean ± SEM from 8 independent experiments. S) CSCs were pre‐labeled with ^13^C‐acetate and cultured for 24 h. Subsequently, exosomes were isolated from this conditioned medium, and the intra‐exosomal levels of ^13^C‐labeled acetyl‐CoA were quantified by UPLC‐MS. T) Intracellular acetyl‐CoA concentrations in CD8^+^ T cells following 24 h exposure to CSC‐derived exosomes. Data presented as mean ± SEM from 6 individuals. U) CD8^+^ T cells cultured for 24 h with CSC exosomes/MVs prior to ex vivo induction of CD103^+^ differentiation. CD8^+^CD103^+^ T cell frequencies were determined by flow cytometry. Shown from 6 individuals. V) Following a 24 h pretreatment of CSCs with the ACLY inhibitor BMS‐303141 (40 µm) and an ACSS2 inhibitor (10 µm), exosomes were isolated from the conditioned medium and co‐cultured with CD8⁺ T cells. The induction of CD8⁺CD103⁺ T cells was then analyzed by flow cytometry. Data are presented as Mean ± SEM from 4 individuals. W,X) Syngeneic LLC‐bearing mice were treated with CD133 antibody‐conjugated NPs loaded with BMS303141, followed by monitoring of CD8^+^CD103^+^ T cell populations and tumor growth. Mean ± SEM from 6 mice in each group. Y) Syngeneic LLC‐bearing mice were pretreated with the M290‐MC‐MMAF, followed by treatment using CD133 antibody‐conjugated NPs loaded with BMS303141. Mean ± SEM from 6 mice in each group. *p < 0.05, **p < 0.01, ***p < 0.001, and ****p < 0.0001 with paired *t*‐test A, F,G, I, K, T, W), unpaired *t*‐test B, E), ANOVA plus Tukey's method J, N, R, U,V, X) and Pearson correlation statistical analysis (Q).

Our characterization of CSC‐T cell interactions revealed that CSCs‐conditioned medium potently inhibited CD103^+^ T cell differentiation (**Figure** **3**A), indicating a cell‐contact independent mechanism. Importantly, CD8^+^ T cells isolated from NSCLC patients similarly exhibited impaired CD103^+^ T cell differentiation (Figure 3B).

To elucidate the mechanism by which CSCs suppress CD103⁺ T cell differentiation, we first targeted canonical immunosuppressive pathways. Specifically, we knocked down TGF‐β or IL‐10 genes, or inhibited IDO synthesis in CSCs, but none of these interventions reversed the inhibitory effect as expected (Figure S9A, Supporting Information). Given our previous study demonstrating abnormal metabolism in CSCs,^[^ ^24^ ^]^ we performed comparative metabolic profiling of CSCs versus non‐CSCs. This analysis revealed significant alterations in lipid metabolism, characterized by enhanced lipogenesis and triglyceride (TG) accumulation in CSCs (Figure 3C,D; Figure S9B, Supporting Information). To explore whether CSCs transfer lipids to T cells, we measured Bodipy staining and TG levels in CD8⁺ T cells with or without CSC co‐culture—no significant changes were observed in T cells (Figure S9C,D, Supporting Information). Furthermore, we pretreated CSCs with inhibitors or shRNAs targeting the top 5 upregulated lipid metabolites (Figure S9E, Supporting Information), then co‐cultured these treated CSCs with CD8⁺ T cells. This approach also failed to block the suppressive effect of CSCs (Figure S9F,G, Supporting Information).

We next quantified intracellular levels of acetyl‐CoA, an essential substrate for lipogenesis, and observed a significant elevation in CSCs (Figure 3E). We found that both CSCs and their culture supernatant—but not non‐CSCs—efficiently increased acetyl‐CoA levels in co‐cultured T cells (Figure 3F,G; Figure S9H, Supporting Information). Consistent with this, when CSCs were fed ^13^C‐acetate, we detected notable ^13^C enrichment in recipient CD8⁺ T cells, providing direct evidence that acetyl‐CoA is transferred from CSCs to T cells (Figure 3H). Of note, acetate treatment alone also upregulated intracellular acetyl‐CoA levels, thereby inhibiting CD103^+^ T cell differentiation (Figure 3I; Figure S9I,J, Supporting Information). Given that acetyl‐CoA is generated either by ATP‐citrate lyase (ACLY) from mitochondrial citrate or by acetyl‐CoA synthetase short‐chain family member 2 (ACSS2) from acetate,^[^ ^29^ ^]^ we blocked its production in CSCs using the ACLY inhibitor BMS303141 in combination with an ACSS2 inhibitor. This treatment abolished the CSC‐mediated suppression of CD103^+^ T cell differentiation (Figure 3J). Furthermore, genetic knockdown of ACLY and ACSS2 within CSCs effectively impeded the effect of CSCs on CD103^+^ T cell differentiation (Figure 3K; Figure S9K, Supporting Information), together with compromised CSC stemness (Figure S9L, Supporting Information). Collectively, these results demonstrate that CSC‐derived acetyl‐CoA critically mediates the suppression of CD103^+^ T cell differentiation.

In line with the impaired CD103^+^ T cell differentiation, exogenous acetate efficiently reduced the protein level of Blimp‐1 in T cells (Figure 3L). Of note, overexpression of Blimp‐1 could abrogate the effect of acetate on CD103^+^ T cell differentiation (Figure 3M,N). We further confirmed that blocking acetyl‐CoA generation in CSCs could rescue the protein level of Blimp‐1 in response to CSCs, a process that could be abrogated by exogenous acetate (Figure 3O). This phenomenon was confirmed by genetic knockdown of ACLY plus ACSS2 within CSCs (Figure 3P). Such findings pinpoint an essential role of CSC acetyl‐CoA in reducing the protein level of Blimp‐1 of T cells, which was validated with single‐cell analyses of tumor tissues from NSCLC patients, showing a negative correlation between acetyl‐CoA generation within CSCs and the frequency of CD8^+^CD103^+^ T cells (Figure 3Q). To exclude the contribution of T‐cell‐intrinsic acetyl‐CoA synthesis to CD103⁺ T cell differentiation, we directly applied ACLY/ACSS2 inhibitors to T cells alone. Our results showed that while these inhibitors reduced CD8⁺CD103⁺ T cell differentiation (likely due to impaired energy supply), Blimp‐1 expression remained unaltered under this condition (Figure S9M,N, Supporting Information).

To investigate the cell‐contact‐independent mechanism by which CSCs suppress CD103⁺ T cell differentiation, we focused on extracellular vesicles (EVs). We found that acetyl‐CoA was highly enriched in the exosome fraction compared to the microvesicle fraction from CSCs (Figure 3R; Figure S10A, Supporting Information). Critically, when CSCs were fed ^13^C‐acetate, we detected ^13^C‐labeled acetyl‐CoA in subsequently isolated exosomes, providing direct evidence for the active packaging of acetyl‐CoA into these vesicles (Figure 3S). This CSC‐derived exosomal acetyl‐CoA was functionally active: only exosomes from CSCs—not from non‐CSCs, nor macrovesicles from CSCs—significantly increased intracellular acetyl‐CoA levels in CD8⁺ T cells and suppressed CD103⁺ T cell differentiation (Figure 3T,U; Figure S10B, Supporting Information). To systematically clarify that it is exosome‐encapsulated acetyl‐CoA (rather than unencapsulated factors or other metabolites) that mediates this effect, we performed a series of validation experiments: we treated exosomes that had undergone multiple verification (including nanoparticle tracking analysis, electron microscopy, and phenotype identification) (Figure S10C–E, Supporting Information) with heat inactivation (Figure S10F, Supporting Information), Proteinase K (with or without detergent) (Figure S10G, Supporting Information), prepared exosome‐depleted conditioned medium (Figure S10H, Supporting Information), and treated CSCs with GW4869 (an EV biogenesis inhibitor) (Figure S10I,J, Supporting Information), and these experiments collectively confirmed that the metabolite encapsulated in exosomes is responsible for inhibiting CD103⁺ T cell differentiation; we also generated CSC‐derived exosomes by pretreating CSCs with BMS303141 plus ACSS2 inhibitor, or inhibitors targeting other lipid metabolites, and only exosomes from CSCs with inhibited acetyl‐CoA production lost the inhibitory activity (Figure 3V; Figure S10K, Supporting Information), further attributing this effect specifically to acetyl‐CoA.

To validate the physiological role of CSC‐derived acetyl‐CoA in suppressing CD103^+^ T cell differentiation, we employed CD133 antibody‐conjugated nanoparticles loaded with the ACLY inhibitor BMS303141 in syngeneic LLC models—this design was based on our finding that ACLY is the primary enzyme mediating acetyl‐CoA synthesis in CSCs (Figure S10L,M, Supporting Information). This targeted inhibition of acetyl‐CoA production in CSCs significantly increased the frequencies of intratumoral CD8^+^CD103^+^ T cells and attenuated tumor progression (Figure 3W–X; Figure S10N, Supporting Information). The effect of such BMS‐NPs on tumor growth depended on CD103^+^ T cells, as their depletion abolished these effects (Figure 3Y). These results conclusively establish CSC‐generated acetyl‐CoA as a critical regulator of CD103^+^ T cell differentiation in the TME. To further clarify the role of CSC‐derived exosomes in promoting non‐CSC tumorigenesis, we subcutaneously co‐implanted PDO‐derived non‐CSCs with or without CSC‐derived exosomes into NSG mice, and concurrently administered autologous PBMCs via intravenous injection. In vivo, co‐implantation of CSC‐derived exosomes significantly suppressed CD8⁺CD103⁺ T cell differentiation (Figure S10O, Supporting Information). Moreover, mice receiving CSC‐derived exosomes exhibited a higher tumorigenesis rate and earlier tumor onset compared to controls (Figure S10P,Q, Supporting Information).

### 2.4 CSC‐Derived Acetyl‐CoA Induces Blimp‐1 Acetylation in T Cells

**Figure 4:**
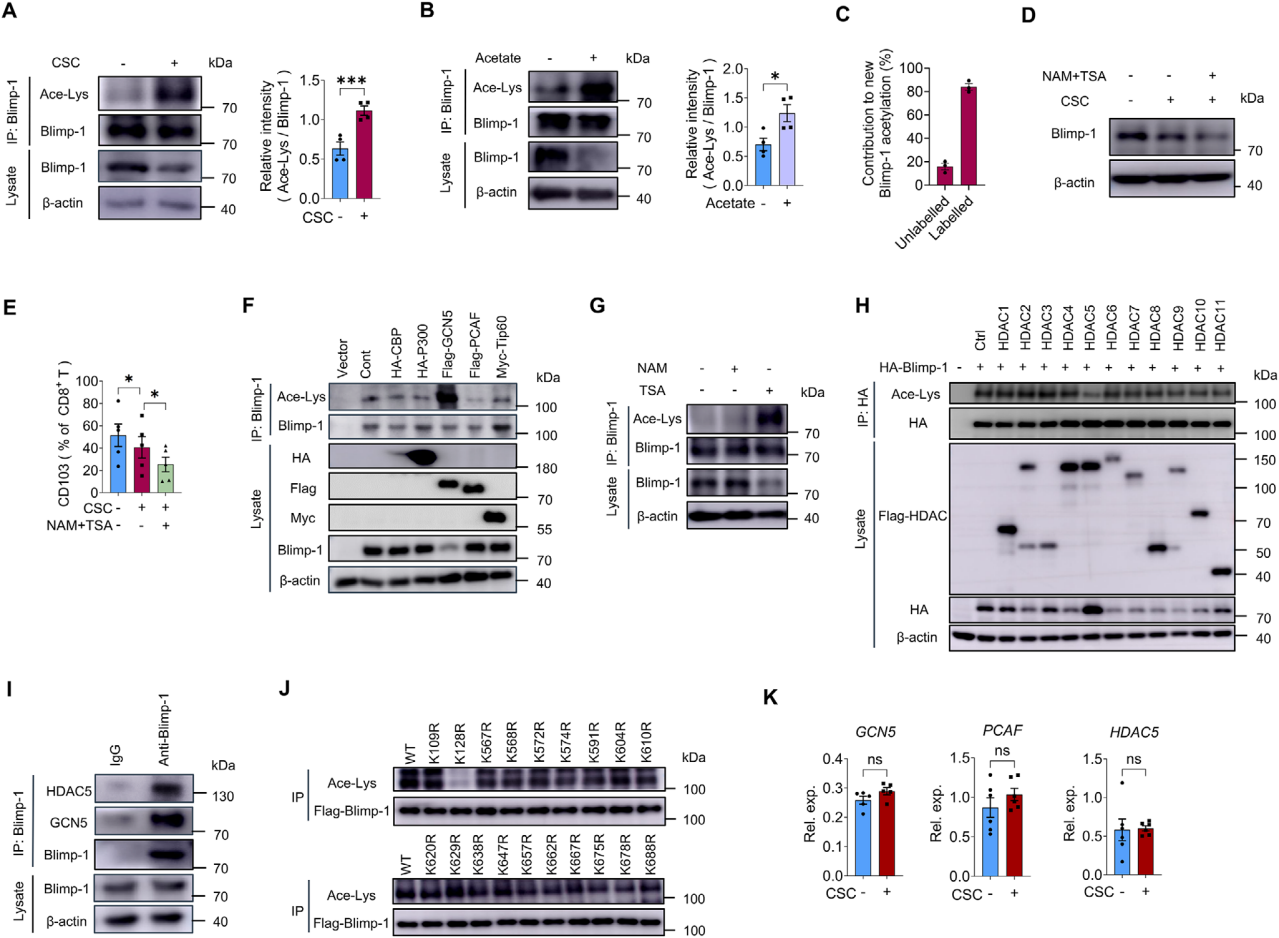
Acetyl‐CoA from CSCs enhances the acetylation of Blimp‐1 in T cells. A) CD8^+^ T cells were cocultured with or without CSCs for 24 h prior to ex vivo induction of CD8^+^CD103^+^ T cells. Endogenous Blimp‐1 acetylation was analyzed by immunoprecipitation in the induced T cell populations. Mean ± SEM from 4 individuals in each group. B) CD8^+^CD103^+^ T cells were induced ± acetate (10 mm), and the acetylation of Blimp‐1 was detected. Mean ± SEM from 4 individuals in each group. C) After co‐culture of ^13^C‐acetate pre‐labeled CSCs with primary CD8⁺ T cells, the T cells were isolated, and the Blimp‐1 was immunoprecipitated for targeted MS analysis. D,E) Immunoblot analysis of Blimp‐1 protein and flow cytometry analysis of CD8^+^CD103^+^ T cells were performed in CSC‐conditioned CD8^+^ T cells treated with or without NAM (2 mm) and TSA (1 µm). Each dot represents one individual. F) Immunoprecipitation analysis of Blimp‐1 acetylation in HEK293T cells that were co‐transfected with Blimp‐1 and the indicated acetyltransferase. G) Immunoprecipitation analysis was performed to assess Blimp‐1 acetylation in induced CD8^+^CD103^+^ T cells that were treated with NAM (2 mm) or TSA (1 µm) for 12 h. H) Immunoprecipitation analysis of Blimp‐1 acetylation was performed in HEK293T cells overexpressing individual deacetylases (HDAC1‐11). I) Co‐immunoprecipitation of endogenous Blimp‐1 with HDAC5 and GCN5 was performed in induced CD8^+^CD103^+^ T cells. J) Immunoprecipitation analysis of Blimp‐1 acetylation in HEK293T cells that were transfected with Flag‐Blimp‐1 or its lysine mutants. K) Acetyltransferase and deacetylase gene expression was examined in induced CD103^+^ T cells after CSC pre‐education. Shown from 5 to 6 individuals. *p < 0.05 and ***p < 0.001 with paired *t*‐test A,B) and ANOVA plus Tukey's method (E).

Acetyl‐CoA serves as the primary acetyl group donor for lysine acetylation. This PTM is catalyzed by lysine acetyltransferases (KATs), which transfer the acetyl group to the ε‐amino side chain of lysine residues—a process reversed by deacetylases.^[^ ^30^ ^]^ We examined Blimp‐1 acetylation in T cells under CSC‐conditioned culture and observed that CSC preincubation enhanced Blimp‐1 acetylation (**Figure** **4**A). Notably, acetate treatment also significantly increased Blimp‐1 acetylation (Figure 4B), suggesting a key role of acetyl‐CoA in CSC‐T cell interactions. To directly confirm that the acetyl groups on Blimp‐1 originated from the transferred CSC‐derived acetyl‐CoA, we performed immunoprecipitation of Blimp‐1 followed by targeted mass spectrometry (MS). Results showed that 80% of the acetyl‐modified peptides from Blimp‐1 were ^13^C‐labeled, verifying that the transferred acetyl‐CoA is functionally used for Blimp‐1 acetylation (Figure 4C). Concurrently, Blimp‐1 protein levels were reduced by both CSC preincubation and exogenous acetate (Figure 4A,B), implying that acetylation may promote Blimp‐1 ubiquitination. Consistent with this, when T cells were preincubated with CSCs in the presence of trichostatin A (TSA, a broad‐spectrum HDAC inhibitor) and nicotinamide (NAM, a SIRT deacetylase inhibitor), we observed further downregulation of Blimp‐1 protein and a decreased frequency of CD103^+^ T cells (Figure 4D,E).

To identify the acetyltransferase responsible for Blimp‐1 modification, we systematically overexpressed common acetyltransferases and found that GCN5 specifically induced Blimp‐1 acetylation (Figure 4F). For deacetylation analysis, healthy T cells were treated separately with TSA or NAM. Only TSA treatment affected Blimp‐1 acetylation and protein levels (Figure 4G),^[^ ^31^ ^]^ implicating HDAC family members in Blimp‐1 regulation. Using a similar screening approach, we identified HDAC5 as the specific deacetylase for Blimp‐1 (Figure 4H). Co‐immunoprecipitation assays confirmed direct interactions between Blimp‐1 and both GCN5 and HDAC5 in T cells (Figure 4I). Furthermore, using site‐directed mutants, we identified K128 as the principal acetylation site on Blimp‐1 (Figure 4J).

To determine whether CSCs influence GCN5 and HDAC5 expression, we compared their mRNA levels in T cells with or without CSC preincubation. Our results showed that CSCs did not alter the expression of these acetyltransferases or deacetylases in T cells (Figure 4K). These findings indicate that CSC‐derived acetyl‐CoA, rather than changes in enzyme expression, drives Blimp‐1 acetylation in T cells.

### 2.5 Protein Acetylation Regulates Blimp‐1 Ubiquitination in T Cells

**Figure 5:**
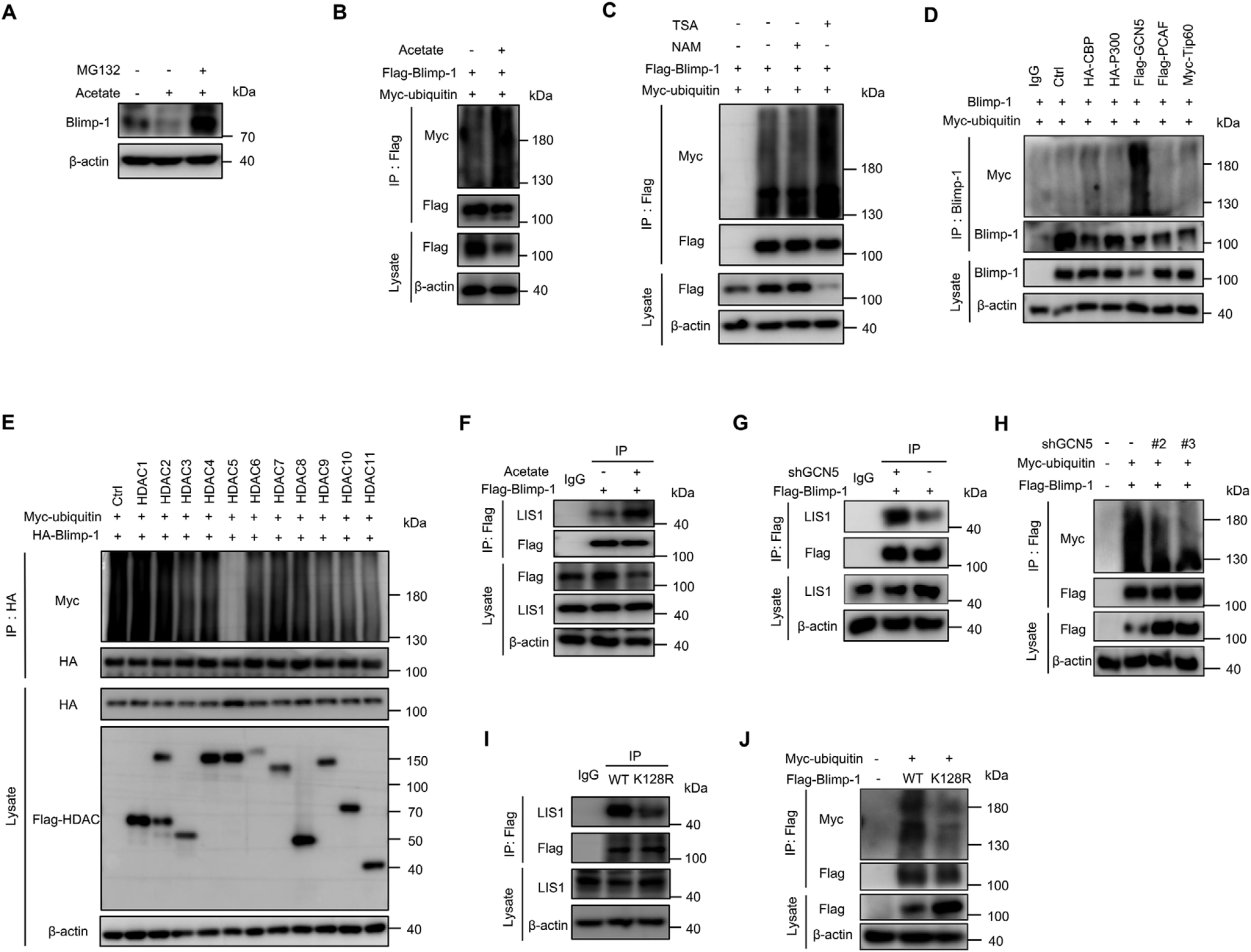
Acetylation of Blimp‐1 drives its ubiquitination. A) CD8⁺CD103⁺ T cells were induced ex vivo with acetate (10 mm), in the presence or absence of MG132 (2 µm) for 4 h. Blimp‐1 protein expression was analyzed by immunoblotting. B) Immunoprecipitation analysis of Blimp‐1 ubiquitination in HEK293T cells treated with or without acetate (15 mm). C) Immunoprecipitation analysis of Blimp‐1 ubiquitination was performed in HEK293T cells co‐transfected with Blimp‐1 and ubiquitin, and treated with or without NAM (4 mm) or TSA (2 µm) for 12 h. D) Immunoprecipitation analysis of Blimp‐1 ubiquitination in HEK293T cells cotransfected with Blimp‐1 and indicated acetyltransferase. E) Immunoprecipitation analysis of Blimp‐1 ubiquitination in HEK293T cells that were co‐transfected with HA‐Blimp‐1 and the indicated deacetylases. F) Co‐immunoprecipitation analysis of Blimp‐1 and LIS1 interaction in HEK293T cells in the presence or absence of acetate (15 mm). G) Co‐Immunoprecipitation analysis of the interactions between Blimp‐1 and LIS1 in HEK293T cells that were co‐transfected with Flag‐Blimp‐1 and shGCN5. H) Immunoprecipitation analysis of ubiquitination of Blimp‐1 in HEK293T cells that were co‐transfected with Flag‐Blimp‐1, Myc‐Ub, and shGCN5. I) Co‐immunoprecipitation analysis of Blimp‐1 (WT or K128R) and LIS1 interaction in HEK293T cells. J) Immunoprecipitation analysis of ubiquitination of Blimp‐1 (WT or K128R) in HEK293T cells that were co‐transfected with Flag‐Blimp‐1(WT or K128R) and Myc‐Ub.

To establish the link between Blimp‐1 acetylation and ubiquitination, we treated T cells with MG132 in the presence of acetate. MG132 effectively rescued the acetate‐induced reduction of Blimp‐1 protein levels (**Figure** **5**A), confirming proteasomal degradation. Consistently, acetate treatment markedly increased Blimp‐1 ubiquitination (Figure 5B). Supporting the role of GCN5 and HDAC5 in this regulation, both TSA treatment and GCN5 overexpression enhanced Blimp‐1 ubiquitination (Figure 5C,D), whereas HDAC5 overexpression reduced it (Figure 5E). Together, these results demonstrate that Blimp‐1 acetylation triggers its ubiquitination and subsequent degradation.

We next investigated the mechanism underlying Blimp‐1 acetylation‐enhanced ubiquitination by transfecting HEK293T cells with Blimp‐1 and treating them with acetate. Co‐immunoprecipitation assays showed that acetate treatment strengthened the interaction between Blimp‐1 and E3 ligase LIS1 (Figure 5F). Accordingly, GCN5 knockdown significantly impaired the Blimp‐1‐LIS1 interaction and reduced Blimp‐1 ubiquitination (Figure 5G,H; Figure S11, Supporting Information). Moreover, mutating K128—the key acetylation site—significantly reduced both the Blimp‐1‐LIS1 interaction and ubiquitination (Figure 5I,J). These results demonstrate that Blimp‐1 acetylation at K128 promotes LIS1 binding, thereby enhancing ubiquitination.

### 2.6 Targeting CSC‐T Cell Interactions to Rescue CD103^+^ T Cell Differentiation and Restrict NSCLC Outgrowth

**Figure 6:**
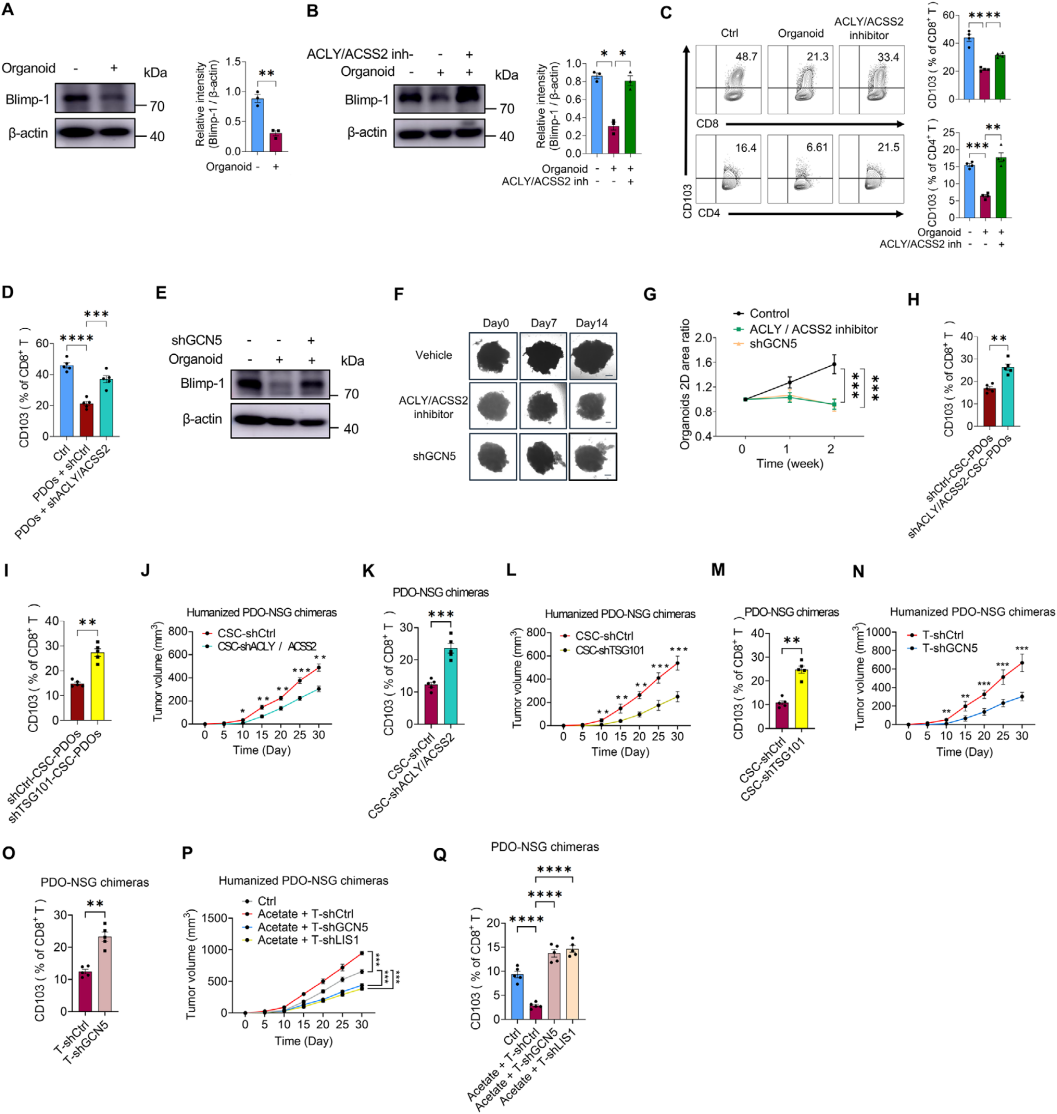
Blocking CSC‐mediated suppression of CD103⁺ T cells to inhibit NSCLC expansion. A) CD8⁺ T cells were preconditioned with PDOs for 24 h, followed by ex vivo induction of CD8⁺CD103⁺ T cells. Immunoblot analysis showed Blimp‐1 expression in induced T cells. Mean ± SEM from 3 individuals in each group. B,C) CD8⁺ and CD4⁺ T cells were co‐cultured for 24 h with PDOs pretreated with or without BMS‐303141 (40 µm) and an ACSS2 inhibitor (10 µm). The T cells were then isolated, activated, and induced to differentiate into CD103⁺ populations. Blimp‐1 protein levels in CD8⁺ T cells and the differentiation of CD8⁺CD103⁺, and CD4⁺CD103⁺ T cells were analyzed by immunoblotting and flow cytometry, respectively. Mean ± SEM from 3 individuals in each group. D) PDOs were transfected with shRNAs targeting ACLY and ACSS2 before the PDO‐PBMC coculture, and then the differentiation of CD8^+^CD103^+^ T cells was detected. Mean ± SEM from 5 individuals in each group. E) CD8^+^ T cells were incubated with PDOs for 24 h and then transfected with shRNAs against GCN5. Blimp‐1 protein in induced T cells was analyzed by immunoblotting. F,G) PDOs were treated with or without BMS‐303141 (40 µm) plus ACSS2 inhibitor (10 µm), then incubated with either control PBMCs or PBMCs pre‐transfected with GCN5 shRNA. PDO outgrowth was subsequently measured. Mean ± SEM from 4 individuals in each group. Scale bars, 200 µm. H,I) CD133‐expressing CSCs were isolated from PDOs, transfected with the indicated shRNAs, and reintroduced into the PDOs for subsequent PDO‐PBMC co‐culture. CD8^+^CD103^+^ T cells were assessed by flow cytometry. Mean ± SEM from 5 individuals in each group. J–M) CD133‐expressing CSCs were isolated from PDOs, transfected with the indicated shRNAs, and reintroduced into the parental PDOs. These reconstituted PDOs were then implanted into NSG mice, which were intravenously injected with patients’ PBMCs. Tumor growth and tumor‐infiltrating CD8^+^CD103^+^ T cells were subsequently analyzed. Mean ± SEM from 5 chimeras in each group. N,O) CD8^+^ T cells isolated from patient‐derived PBMCs were transfected with the indicated shRNAs and reintroduced into the PBMC population for immune reconstitution of PDO‐NSG chimeric mice. Tumor growth and tumor‐infiltrating CD8^+^CD103^+^ T cells were subsequently analyzed. Mean ± SEM from 5 chimeras in each group. P,Q) CD8^+^ T cells isolated from patient‐derived PBMCs were transfected with the indicated shRNAs and reintroduced into the PBMC population for immune reconstitution of PDO‐NSG chimeric mice treated with or without acetate. Tumor growth and tumor‐infiltrating CD8^+^CD103^+^ T cells were subsequently analyzed. Mean ± SEM from 5 chimeras in each group. **p < 0.01, ***p < 0.001, ****p < 0.0001 with paired *t*‐test H,I, K, M, O) and ANOVA plus Tukey's method D, G, J, L, N, P,Q).

Although murine tumor models have significantly contributed to our understanding of tumor biology and played a pivotal role in anti‐tumor therapy development, drugs demonstrating efficacy in murine studies have frequently failed to translate to human tumors. In contrast, PDOs exhibit drug response profiles that closely mirror those observed in the corresponding patients for both conventional and investigational agents.^[^ ^32^ ^]^ Therefore, we cultured PBMCs from NSCLC patients with matched PDOs to simulate in vivo conditions and evaluated the translational application of targeting CSC‐mediated Blimp‐1 acetylation. While NSCLC PDOs could reduce the protein level of Blimp‐1 in interacting T cells (**Figure** **6**A), pretreatment of PDOs with BMS303141 and ACSS2 inhibitor effectively restored both Blimp‐1 expression and CD103^+^ T cell differentiation (Figure 6B,C). This effect was confirmed using PDOs pre‐transfected with ACLY‐ and ACSS2‐targeting shRNAs before PBMC co‐culture, which similarly enhanced CD103^+^ T cell differentiation (Figure 6D). Furthermore, genetic knockdown of GCN5 in T cells also restored Blimp‐1 protein levels in PDO‐conditioned CD103^+^ T cells (Figure 6E). Importantly, targeting of acetyl‐CoA synthesis in PDOs and GCN5 in T cells significantly inhibited NSCLC PDO outgrowth (Figure 6F,G). These findings demonstrate that disrupting CSC‐mediated Blimp‐1 acetylation represents a promising therapeutic strategy for NSCLC treatment.

To validate the translational potential of targeting CSC‐derived acetyl‐CoA, we isolated CD133^+^ CSCs from NSCLC PDOs and transfected them with ACLY‐ or ACSS2‐targeting shRNAs. These genetically modified CSCs were then recombined with CD133^−^ cells to reconstruct PDOs for use in the PDO‐PBMC co‐culture system. Our results demonstrated that inhibiting acetyl‐CoA production specifically in CSCs successfully restored CD103^+^ T cell differentiation within the PDOs (Figure 6H). Further, when CD133^+^ CSCs from PDOs were pre‐transfected with shRNA targeting the multivesicular body (MVB) protein TSG101 to impede their exosome secretion, such PDOs were unable to suppress the differentiation of CD103^+^ T cells (Figure 6I). Thus, blocking CSC‐derived exosomal acetyl‐CoA appears promising for increasing CD103^+^ T cells within the reconstituted TME.

To validate our findings in vivo, we implanted single‐cell suspensions from PDOs containing ACLY‐ and ACSS2‐knockdown CSCs into immunodeficient NSG mice, followed by immune reconstitution with the same patient‐derived PBMCs. Our results demonstrated that lentiviral‐mediated knockdown of ACLY and ACSS2 in CSCs significantly reduced tumor growth while increasing the frequency of tumor‐infiltrating CD8^+^CD103^+^ T cells (Figure 6J,K). Similarly, TSG101 knockdown in CSCs also attenuated tumor growth and enhanced CD8^+^CD103^+^ T cell infiltration (Figure 6L,M). Furthermore, when T cells from PBMCs were pre‐transfected with lentiviral shRNA targeting GCN5 prior to immune reconstitution of the humanized PDO chimeras, GCN5 knockdown in T cells efficiently downregulated tumor growth in NSG mice, along with increased CD103^+^ T cells (Figure 6N,O). Finally, we demonstrated that while exogenous acetate treatment promoted the tumor growth in PDO‐NSG chimeras, this process could be abrogated by genetic knockdown of either GCN5 or LIS1 in T cells, accompanied by elevated frequencies of CD103^+^ T cells (Figure 6P,Q). Collectively, these findings strongly demonstrate that targeting CSC‐derived exosomal acetyl‐CoA, along with acetylation and ubiquitination of the Blimp‐1 protein in T cells, may hold promise in restoring CD103^+^T cell differentiation and restraining the progression of human NSCLC.

## 3 Discussion

While CD103^+^ T cells are well‐acknowledged to be crucial in protecting against infectious diseases, recent studies have shown that they play a critical role in antitumor immunity. However, the differentiation of CD103^+^ T cells in TME is largely undefined, despite the fact that the relationship between the CD103^+^ T cell ratio and tumor prognosis has been highlighted. In this study, we uncover that exosome‐encapsulated acetyl‐CoA from CSCs promotes the acetylation of Blimp‐1 protein in interacting T cells. The acetylated Blimp‐1 attracts the E3 ligase LIS1, resulting in enhanced ubiquitination of Blimp‐1 and consequently impaired CD103^+^ T cell differentiation. Determining the process by which CSCs control CD103^+^ T cell differentiation may offer a novel target for immunotherapy. It is evident that preventing the production of CSC acetyl‐CoA and the acetylation of Blimp‐1 are critical for the preservation of CD8^+^CD103^+^ T cells and tumor elimination.

In recent years, research has made significant progress in identifying CD103^+^ T cells in a variety of tissues, including skin, lung, gut, liver, and brain.^[^ ^27^, ^33^, ^34^, ^35^, ^36^ ^]^ As CD103 promotes the long‐term retention and survival of T cells, CD103‐positive cells are frequently referred to as tissue‐resident memory T cells.^[^ ^27^, ^37^, ^38^ ^]^ TGF‐β makes unquestionable contributions in local contexts since it is a primary driver of CD103 expression.^[^ ^26^ ^]^ CD103^+^ T cells inhibit tumor relapse by mediating second challenges more quickly than circulating memory T cells. Upon re‐stimulation, CD103^+^ T cells generate elevated levels of cytokines associated with cell cytotoxicity.^[^ ^20^ ^]^ Despite enhanced inhibitory receptor expression, CD103^+^ T cells keep their polyfunctionality in response to anti‐PD‐1 therapy.^[^ ^39^ ^]^ Of note, CD103^+^ T cells were negatively associated with circulating tumor cells, supporting their suppressive function against tumor metastasis.^[^ ^40^ ^]^ Thus, CD103^+^ T cells are an appealing target for oncology due to their tissue selectivity and improved responsiveness to immunotherapy. In this study, we demonstrate that NSCLC CSCs selectively impair the differentiation of CD103^+^ T cells, indicating a potential unique engagement between CSCs and CD103^+^ T cell precursors. Otherwise, those T cells may exert an intrinsic dependency on acetyl‐CoA for differentiation. However, the precise underlying mechanisms remain unclear.

CSCs are well‐established drivers of tumor recurrence, metastasis, multidrug resistance, and radiation resistance,^[^ ^22^ ^]^ making them a major contributor to treatment failure in cancer. Given the crucial role of CSCs in cancer management, the molecular bases underpinning their stemness have long been an active area of investigation. The Wnt/β‐catenin, together with Hedgehog signaling, have important functions in stem cell maintenance and tumorigenesis. IL‐6/JAK/STAT3 and TGF‐β/Smad signaling induce the proliferation and metastasis of lung CSCs.^[^ ^41^ ^]^ The TME is essential to the self‐renewal of CSCs, inducing angiogenesis, tumor invasion, and metastasis. CSCs adjacent to endothelial cells differentiate into cancer vascular stem cells and participate in angiogenesis. Further, increasing evidence has shown that the ECM, including fibronectin, vimentin, collagen, and proteoglycan bind to cytokines such as FGF in the TME to promote cell proliferation and chemotherapeutic resistance and increase the expression of CSC‐related markers.^[^ ^41^ ^]^ Of note, emerging data has demonstrated that metabolic reprogramming plays a critical role in determining CSC fate. Specifically, we have demonstrated that robust mitophagy of CSCs results in increased content of mitochondrial DNA (mtDNA) in the organelle lysosome, which acts as an endogenous ligand for Toll‐like receptor 9 (TLR9) that interacted with Notch1, enhancing Notch1‐AMPK signaling. Meanwhile, Notch1 hyperactivity may promote mTOR activation and subsequent lipogenesis, leading to elevated expression of OPA1 and mitochondrial fusion.^[^ ^24^, ^42^ ^]^ In consequence, CSCs maintain optimal mitochondrial function that licenses the stemness for tumor progression. While the potential involvement of aberrant mitochondrial dynamics in CSCs in T cell differentiation remains unexplored, our current study demonstrates that acetyl‐CoA—a key lipogenic carbon source in CSCs—plays a critical role in CSC‐mediated inhibition of CD103^+^ T cell differentiation.

Notably, our findings demonstrate that acetyl‐CoA is transferred from CSCs to T cells via an exosome‐dependent mechanism. While three primary mechanisms of exosomal signal transduction are recognized (endocytosis, membrane fusion, and ligand‐receptor interaction),^[^ ^43^ ^]^ the first two appear less likely to be dominant in our context. First, T cells have limited endocytic capacity, making significant exosome uptake improbable.^[^ ^44^ ^]^ Second, while membrane fusion events (as assessed by PKH67 labeling) were indeed frequent, considering the dominant non‐CSCs in tumor tissues, their frequency between CSCs and T cells was consistently lower than that between non‐CSCs and T cells. This suggests that membrane fusion, while present, may not be the primary or most efficient pathway for CSC‐derived exosomes. Therefore, we propose the third mechanism: a specific ligand‐receptor interaction. This is supported by the established concept that exosomes can exhibit tropism for specific cell types.^[^ ^43^ ^]^ Given that CSC‐derived exosomes carry unique molecular cargo^[^ ^43^ ^]^ and that our data show CSCs specifically affect CD103⁺ T cell precursors without broadly impacting other immune subsets, we hypothesize these exosomes mediate their inhibitory effect via targeted binding to specific receptors on CD103⁺ precursor T cells. However, it should be noted that acetyl‐CoA can also be derived from alternative pathways, such as intermediates (e.g., citrate, acetate) or shuttle systems (e.g., citrate‐pyruvate),^[^ ^45^ ^]^ which deserve successive studies during the interactions between CSCs and T cells.

Blimp‐1 is named because of its importance in plasma cell differentiation, and it also affects the homeostasis and function of CD4^+^ and CD8^+^ T cells. The plasma cells in *PRDM1* ^−/−^ mice were almost absent during the immune response.^[^ ^46^ ^]^ During the antigenic response phase, Blimp‐1 levels in T cells are significantly upregulated. And mice expressing a mutant Blimp‐1 develop a fatal multiorgan inflammatory disease caused by T cell accumulation.^[^ ^47^ ^]^ Studies in recent years have shown that Blimp‐1 can bind with the KLF2 locus to repress the expression of tissue egress gene KLF2, which contributes to the formation of CD103^+^ T cells.^[^ ^14^, ^48^ ^]^ Herein, we reveal that CSCs impair CD103^+^ T cell differentiation through an acetyl‐CoA‐dependent mechanism involving Blimp‐1 regulation. As the essential substrate for lysine acetylation, acetyl‐CoA mediates post‐translational modifications of metabolic enzymes, signaling proteins, and transcription factors. While five major lysine acetyltransferases (KATs: CBP, p300, GCN5, PCAF, and TIP60) catalyze most acetylation events, HDACs and sirtuins counterbalance this process.^[^ ^30^ ^]^ In this study, we identify GCN5 and HDAC5 as the key enzymes modulating Blimp‐1 acetylation in T cells. Critically, acetyl‐CoA promotes Blimp‐1 acetylation, which destabilizes the Blimp‐1 protein and consequently suppresses CD103^+^ T cell differentiation.

Proteins are frequently subject to multiple post‐translational modifications (PTMs) that exhibit reciprocal regulatory interactions, a phenomenon commonly termed PTM crosstalk.^[^ ^49^ ^]^ PTM crosstalk can integrate diverse signals and vastly increase their regulatory potential.^[^ ^30^, ^50^ ^]^ In this study, we uncover that the ubiquitination of Blimp‐1 can be increased by acetylation of Blimp‐1 at K128, which promotes the recruitment of LIS‐1 and thereby enhances ubiquitination at multiple sites. We provide compelling evidence that the ubiquitination of Blimp‐1 is accompanied by the acetylation of it in CSCs‐conditioned CD8^+^CD103^+^ T cells. Given that lysine acetylation and ubiquitination are mutually exclusive modifications, K109, K568, K574, K610, K667, K675, and K688 are likely involved in Blimp‐1 ubiquitination. However, the regulatory mechanism by which K128 acetylation influences ubiquitination at other residues remains unexplored. Notably, while LIS1 is classically recognized for its role in microtubule regulation,^[^ ^51^ ^]^ its function as an E3 ligase remains unexplored to date. We speculate there may be a potential link between LIS1's E3 ligase activity and its well‐established role in microtubule/dynein regulation—particularly relevant given that the motility, shape maintenance, and cellular integrity critical to CD103⁺ T cells are closely tied to microtubule dynamics.^[^ ^52^ ^]^

PDOs have recently been established for various cancer types, including colon carcinoma, hepatocellular carcinoma, pancreatic cancer, lung cancer, and prostate cancer.^[^ ^53^ ^]^ Importantly, PDOs represent an efficient and economical platform that accurately recapitulates disease characteristics.^[^ ^32^, ^53^ ^]^ Drug sensitivity testing using PDOs has demonstrated high concordance with responses observed in the primary tumor.^[^ ^54^ ^]^ Herein, we demonstrate that both genetic ablation and small‐molecule inhibition of acetyl‐CoA synthesis in PDOs can rescue CD103^+^ T cell differentiation, thereby increasing the anti‐tumor immune response and impeding the tumor progression. Crucially, we rigorously validate the translational relevance of targeting CSC‐derived exosomal acetyl‐CoA, alongside the acetylation and ubiquitination of Blimp‐1 in T cells, utilizing the humanized PDO‐NSG chimeras. Such findings provide promising targets for NSCLC management in clinical practice.

In summary, CSCs play a crucial role by transferring exosomal acetyl‐CoA into T cells, thereby promoting the acetylation of the Blimp‐1 protein. This process enhances the physical interaction between Blimp‐1 and the E3 ligase LIS1, leading to the ubiquitination of Blimp‐1. Consequently, Blimp‐1 ubiquitination inhibits the differentiation of CD103^+^ T cells in the TME. Targeting acetyl‐CoA in CSCs and Blimp‐1 acetylation/ubiquitination in T cells emerges as a promising approach to restore CD103^+^ T cell differentiation and restrict tumor outgrowth. These findings unveil a novel immune‐modulatory function of CSCs in the TME, offering innovative therapeutic strategies for cancer management.

## 4 Experimental Section

### Patients

In total, 91 NSCLC patients and 229 healthy individuals were recruited in this study. Patient characteristics were summarized in Table S1 (Supporting Information). After obtaining informed consent, peripheral blood and tumor tissue samples were collected to study the NSCLC‐instructed differentiation of T cells. Experiments involving human samples were conducted in compliance with Declaration of Helsinki. All the studies were approved by the Ethics Committee of Soochow University (ECSU‐2019000207).

### Patient‐Derived Organoids (PDOs)

PDOs were generated as previously described.^[^ ^24^ ^]^ In brief, the resected tumors from NSCLC patients were minced into ≈0.5–1 mm diameter pieces using fine dissection scissors. Tumor pieces were incubated with RBC lysis buffer (Solarbio, R1010) under gentle rotation for 5 min at room temperature to lyse contaminating RBCs. Then, tumor pieces were distributed in ultra‐low attachment 6‐well culture plates (Corning) with 4 mL per well of PDO medium containing 50% DMEM/F12 (Corning), 50% Neurobasal (Gibco), 1× GlutaMax (Gibco), 1× NEAAs (Gibco), 1× PenStrep (Beyotime, C0222), 1× N21 supplement (R&D systems, AR008), 2‐mercaptoethanol (Thermo Fisher Scientific), and insulin (2.5 mg mL^−1^, Beyotime) and placed on an orbital shaker rotating at 120 rpm within a 37 °C, 5% CO2, 90% humidity sterile incubator. Roughly 75% of the medium was changed every 48 h. PDOs were accessed by immunostainings of PanCK and CD31, together with H&E staining.

### Tumor Spheres and Adherent Cells

Tumor spheres were enriched and used as CSCs as previously described.^[^ ^24^ ^]^ Briefly, human NSCLC cell lines A549/H1299/Calu‐1 from ATCC were maintained in DMEM (Corning) supplemented with 10% FBS (Sigma). Cell suspensions were plated (10 000 cells/well) in 6‐well ultra‐low attachment plates (Corning) in a defined serum‐free medium composed of DMEM/F‐12 (Corning), 1×B‐27 (Thermo Fisher Scientific), EGF (20 ng mL^−1^, Novoprotein), bFGF (20 ng mL^−1^, Novoprotein), insulin (2.5 mg mL^−1^, Beyotime), penicillin/streptomycin (50 units mL^−1^, Beyotime). The adherent cells were used as non‐CSCs.

### T Cell Purification

Peripheral blood mononuclear cells (PBMCs) were isolated by gradient centrifugation with Lymphocyte Separation Medium (DAKEWEI). CD4^+^/CD8^+^ T cells were purified from fresh PBMCs, using an EasySep Human CD4 and CD8 T Cell isolation Kit (STEMCELL Technologies, 17912; STEMCELL Technologies, 17953). The cell purity was consistently over 95% and cells were cultured in RPMI 1640 (Corning) supplemented with 10% FBS (Sigma).^[^ ^55^ ^]^

### NSCLC‐T Cell Interaction

CD4^+^/CD8^+^ T cells were pre‐incubated with CSCs, non‐CSCs, or single cells digested from organoids at a ratio of 1:1, 1:5, and 5:1 for 24 h, followed by activation with anti‐CD3/CD28 beads (Gibco) combined with TGF‐β (10 ng mL^−1^) for 72 h. The differentiation of T cells was detected by the surface molecule CD103 using flow cytometry.^[^ ^26^, ^56^ ^]^

### Humanized NSCLC Chimeras

Immune‐deficient B‐NSG mice (Biocytogen, Beijing) at 6 weeks of age were housed under specific pathogen‐free conditions, implanted subcutaneously with single cells of NSCLC PDOs (2 × 10^6^ cells) into the flank, and immune reconstituted through intravenous injection with PBMCs (10 × 10^6^ cells) from the same clinical patients. For some experiments, CD133‐expressing CSCs from PDOs were isolated and transfected with lentiviral shRNAs targeting ACLY and ACSS2, as well as TSG101, prior to tumor challenge. Otherwise, T cells from PBMCs were isolated and transfected with either lentiviral GCN5 shRNA or LIS1 shRNA before the immune reconstitution. For acetate treatment, chimeras were given sodium acetate (Sigma) in sterile drinking water at a final concentration of 200 mm for 4 weeks, and no difference in water intake was found between different groups. Tumor‐infiltrating CD103^+^ T cells and tumor volumes were calculated as previously described. Tumor volume was calculated (using the formula: [length × width^2^]/2) to monitor tumor initiation and growth. Experiments with NSG mice were performed following the ARRIVE guidelines and approved by the Ethics Committee of Soochow University.

### Syngeneic LLC Model

Male C57BL/6 mice (Laboratory Animal Center of Soochow University) aged 6–8 weeks were subcutaneously injected with 100 µL of LLC cell suspension (5 × 10^5^ cells per mouse) into their right flanks. Post‐injection, the mice were randomly assigned to either the control group or the treatment groups receiving BMS‐303141@aCD133 and DAPT@aCD133 nanoparticles. Subsequently, CD133^+^ CSCs, CD8^+^CD103^+^ T cells, and tumor growth were monitored over a period of 3 weeks. All experiments were performed following the ARRIVE guidelines and approved by the Ethics Committee of Soochow University.

### Reagents and Transfections

The following chemicals were used in this study: sodium acetate (Sigma–Aldrich, 241245), MG132 (MedChemExpress, HY‐13259), bafilomycin A1 (MedChemExpress, HY‐100558), BMS‐303141 (MedChemExpress, HY‐16107), ACSS2 inhibitor (Selleck, S8588), nicotinamide (MedChemExpress, HY‐B0150), trichostatin A (MedChemExpress, HY‐15144), Navoximod (MedChemExpress, HY‐18770B), ML‐193 (MedChemExpress, HY‐110125), Fumonisin B1 (MedChemExpress, HY‐N6719), and FSG67 (MedChemExpress, HY‐112489).

For T cell transfection, primary human T cells were electroporated using the Amaxa Human T Cell Nucleofector Kit (Lonza) following established protocols.^[^ ^57^ ^]^ HEK293T cell transfections were performed using polyethylenimine (PEI) according to the manufacturer's instructions. All shRNA constructs (shBlimp‐1, shACLY, shACSS2, shTSG101, shGCN5, shLIS1, shIL10, shTGFB1, shCRLS1, shCLN5, and control shRNA) and plasmids were obtained from MiaoLing Plasmid Platform. For lentivirus production, HEK293T cells were co‐transfected with the respective shRNAs together with packaging plasmids (psPAX2 and pMD2.G). Viral supernatants were collected 48 h post‐transfection, concentrated by centrifugation, and stored as aliquots at −80 °C until use. All experimental procedures were strictly followed the manufacturers' recommended protocols.^[^ ^58^ ^]^

### Flow Cytometry

For cell surface staining, APC‐Cy7 anti‐human CD4 (BioLegend, 317450), PE‐Cy7 anti‐human CD8 (BD Biosciences, 557746), APC anti‐human CD8 (Invitrogen, 17‐0088‐42), Percyp5.5 anti‐human CD45 (Invitrogen, 45‐0451‐82), APC anti‐human CD69 (BioLegend, 985206), FITC anti‐human CD103 (BioLegend, 350204), PE anti‐human CD103 (BioLegend, 350206), FITC anti‐human CD49a (BioLegend, 328308), 660 anti‐human S1PR1 (Invitrogen, 50‐3639‐42), FITC anti‐human CD279 (PD‐1) (Invitrogen, 11‐9969‐41), APC anti‐human TIM3 (Invitrogen, 17‐3109‐41), PE anti‐human CD45RO (BioLegend, 304206), APC anti‐human CD62L (Invitrogen, 17‐0629‐42), FITC anti‐human CD45RA (Invitrogen, 11‐0458‐42), APC anti‐human KLRG1 (Invitrogen, 17‐9488‐42), PE anti‐human CD127 (Invitrogen, 12‐1278‐42), FITC anti‐human CD11c (Invitrogen, 11‐0116‐42), APC anti‐human CD86 (BioLegend, 305412), 488 anti‐human CD68 (ABclonal, A22514) and APC anti‐human CD206 (Invitrogen, 17‐2069‐42) were used for 45 min at 4 °C. For intracellular staining, cells were fixed with Polyoxymethylene (Biosharp, BL539A) and permeabilized with Perm Buffer III (BD Biosciences, 558050) and incubated with following antibodies: FITC anti‐human IFN‐γ (Biolegend, 502528), PE anti‐human Granzyme B (Biolegend, 396408), PE anti‐human TCF1 (Biolegend, 655207), 594 anti‐human TOX (Biolegend, 682604), APC anti‐human Annexin V (BioGems, 62700‐80), 647 anti‐human T‐bet (BioLegend, 644804), PE anti‐human GATA3 (BioLegend, 653804), APC anti‐human ROR gamma (t) (Invitrogen, 17‐6988‐82), PE‐Cy7 anti‐human FOXP3 (Invitrogen, 25‐4777‐42), PE anti‐human CD133 (Invitrogen, 12‐1338‐42), FITC anti‐mouse CD45 (BioLegend, 103108), APC anti‐mouse CD8 (Invitrogen, 17‐0081‐82), PE anti‐mouse CD103 (Invitrogen, 12‐1031‐82) and PE‐conjugated Pan‐CK (Abcam, ab7753). Cells were stained for 45 min at 4 °C in the dark, and flow cytometry analysis using a Canto II (BD Biosciences). For multicolor flow cytometry, compensation controls were set up using single‐stained samples. Data were analyzed with FlowJo software. The gating strategy was as follows: First, the main cell population was identified on a forward scatter area (FSC‐A) versus side scatter area (SSC‐A) plot to exclude debris. Single cells were then selected from this population using forward scatter height (FSC‐H) versus FSC‐A. Subsequent gates were applied to identify specific immune lineages.^[^ ^56^ ^]^

For patient‐derived organoid (PDO) processing, single cells were obtained after digestion with 300 U mL^−1^ collagenase IV (Worthington, LS004210) at 37 °C for 30 min, followed by three 5‐min washes in RPMI‐1640 (Corning) containing 10% FBS (Sigma). Dissociated cells were stained with APC‐conjugated anti‐human CD133 (Biorbyt, orb99113) for 45 min at room temperature, and viable cells were sorted using a BD FACS Influx system.^[^ ^24^ ^]^

### Immunofluorescence

For tissue staining, tissue slides were placed at room temperature for 60 min and fixed in ice‐cold acetone for 20 min, followed by washing in 1× PBST (0.05% Tween‐20). Staining procedures followed previously published protocols. Tissue sections were incubated with primary antibodies, anti‐human CD31 (Abcam, ab281583), anti‐human PanCK (Abcam, ab7753), anti‐human CD133 (Sigma–Aldrich, ZRB1013), and anti‐human CD103 (Proteintech, 65047) overnight at 4 °C. After washing, slides were incubated with secondary antibodies DyLight 649 (Abbkine, A23610), Dylight 488 (Abbkine, A23220), 647 goat anti‐mouse IgG (Invitrogen, A21235), and 555 goat anti‐rabbit IgG (Invitrogen, A21428) for 1 h. Cell nuclei were counterstained with Hoechst (Yeasen, 40731ES10) for 15 min at room temperature. Images were visualized with confocal microscopy (Nikon) and deconvolved with ImageJ software (NIH).^[^ ^24^ ^]^

### Real‐Time PCR

For RNA isolation, cells were lysed using RNAiso plus (Takara). cDNA was synthesized from 1 µg of total RNA using RT SuperMix according to the manufacturer's protocol. Quantitative PCR (qPCR) was performed using SYBR Green SuperMix. mRNA expression levels were calculated using 2^–ΔCt^. Primer sequences are provided in Table S2 (Supporting Information).^[^ ^42^, ^56^ ^]^

### Immunoblot and Immunoprecipitation

Immunoblotting and immunoprecipitation were performed as previously described.^[^ ^42^, ^56^ ^]^ In brief, cells were lysed in ice‐cold buffer (150 mm NaCl, 50 mm Tris‐HCl, 10% glycerol, 0.5–1% Triton X‐100, 100 µm PMSF) for 30 min at 4 °C. Lysates were clarified by centrifugation (12 000 rpm, 15 min, 4 °C), and supernatants were collected for subsequent analyses. For immunoblotting, proteins were resolved by SDS‐PAGE and transferred to membranes. For immunoprecipitation, clarified lysates were incubated with specific antibodies and protein A/G beads overnight at 4 °C. Immunoprecipitated complexes were washed and analyzed by immunoblotting with designated antibodies. The antibodies with dilutions were as follows: anti‐β‐actin (Santa Cruz Biotechnology, sc‐47778, 1:1000), anti‐Blimp‐1 (Santa Cruz Biotechnology, sc‐66015, 1:500; Cell signal technology, C14A4, 1:1000; Abcam, ab119401, 1:1000), anti‐Smad3 (ZEN BIO, R22774, 1:1000), anti‐p‐Smad3 (Santa Cruz Biotechnology, sc‐517575, 1:1000), anti‐Hobit (Invitrogen, PA1‐30046, 1:1000), anti‐RUNX1 (Abclonal, A2055, 1:1000), anti‐RUNX3 (Abclonal, A7303, 1:1000), anti‐Notch1 (Cell Signaling Technology, 3608, 1:1000), anti‐Eomes (Santa Cruz Biotechnology, sc‐293481, 1:1000), anti‐LAMP1 (Abcam, ab25630, 1:1000), anti‐PAFAH1B1 (Abclonal, A3696, 1:1000), anti‐ubiquitin (Abclonal, A19686, 1:1000; Santa Cruz Biotechnology, sc‐8017, 1:500), anti‐Flag (Abclonal, AE063 1:1000), anti‐HA (Abclonal, AE008, 1:1000), anti‐Myc (Abmart, M200025, 1:1000), anti‐ACLY (Abclonal, A3719, 1:1000), anti‐ACSS2 (Abclonal, A12334, 1:1000), anti‐Acetyl‐Lysine (Abclonal, A2391, 1:1000), anti‐GCN5 (Abclonal, A2224, 1:1000), anti‐HDAC5 (Abclonal, A7189, 1:1000), anti‐CD9 (Abclonal, A1703, 1:1000), anti‐CD63 (Santa Cruz Biotechnology, sc‐365604, 1:1000), anti‐TSG101 (Abclonal, A1692, 1:1000), anti‐Calnexin (Abclonal, A4846, 1:1000), anti‐Flag magnetic beads (Selleck, B26102), anti‐HA magnetic beads (Selleck, B26202) and Protein A/G magnetic beads (Selleck, B23202).

### Processing of scRNA‐seq Data

The single‐cell sequencing data for NSCLC patients were sourced from the GEO database (GSE148071, GSE131907, GSE119911). Malignant and non‐malignant cells, as defined in the original studies, were analyzed separately. Cells expressing fewer than 200 genes or more than 5000 genes were excluded. Additionally, cells with over 30 000 UMIs or mitochondrial content exceeding 30% were filtered out, and the remaining cells were retained for downstream analysis. To address batch effects, the Harmony method was employed. Using Seurat 4.1, the expression matrices were first normalized with the NormalizeData and ScaleData functions. Subsequently, the top 2000 variable genes were identified with the FindVariableFeatures function and principal component analysis was conducted. The first 20 principal components and a resolution of 1.0 were used with the FindClusters function to generate 41 cell clusters. To characterize these clusters, cancer cells were identified using EPCAM, EGFR, TP63, MYC, and SOX2^[^ ^59^, ^60^, ^61^, ^62^, ^63^ ^]^ from the malignant cell cluster, and CD8^+^ T cells were identified using CD8A, CD3E, and CD3D from the non‐malignant immune cell compartment. Within the cancer cell population, CSC subpopulations were further identified by the markers PROM1 and ALDH1A1.^[^ ^64^ ^]^ Similarly, CD8^+^ T cells were subclassified into CD8^+^CD103^+^ T cells using ITGAE and ZNF683.^[^ ^65^ ^]^ Pearson's correlation coefficients between the percentages of CD8^+^CD103^+^ T cells and CSC cells were calculated and visualized in R. Additionally, acetyl‐CoA scores were calculated based on relative gene expressions, including DLAT, DLD, DIP2A, MLYCD, MPC2, ACAT1, ACLY, PDHA1, PDHA2, PDHB, PDK1, PDK2, PDK3, PDK4, ACSS2, PPCS, PDHX, ACSS1.^[^ ^66^, ^67^, ^68^ ^]^ Comparisons were made between the acetyl‐CoA scores of CSC cells and the percentage of CD8^+^CD103^+^ T cells. In our analysis, statistical outliers defined by extremely low values in CD8⁺CD103⁺ T cell percentage, CSC abundance, or acetyl‐CoA score were excluded, as these samples were considered biologically unrepresentative and could compromise the integrity of the analysis.

### RNA‐Seq and Metabolomic Analyses

The GIGA Genomics Facility (BioNovoGene) was employed for RNA‐Seq on tumor spheres and adherent cells. Metabolomic analyses of lipids in tumor spheres and adherent cells were performed by Metware Biotechnology. Those data were archived in the National Genomics Data Center under the accession number PRJCA006881. For the RNA‐Seq procedure, 1–2 µg of total RNA from each sample was utilized for library preparation using the KAPA Stranded RNA‐Seq Library Prep Kit (Illumina). The raw sequencing reads underwent a rigorous quality control process, which involved trimming of adapter sequences and subsequent filtering of reads with an average quality score falling below the threshold of Q20. This step ensured the generation of clean, high‐quality data for further analysis. Alignment of the cleaned sequencing reads to the human reference genome was achieved using HISAT2 with default settings. The read count expression values were quantified utilizing HTSeq. Differential expression analysis was carried out with DESeq, wherein genes exhibiting an absolute fold change greater than 2 and an adjusted P value less than 0.05 were deemed statistically significant.

### Isolation of Exosomes

Exosomes were isolated from the culture medium of CSCs, non‐CSCs, and organoids using differential ultracentrifugation. Briefly, the medium was sequentially centrifuged at 300 × g for 10 min (to remove cells), 10 000 × g for 20 min (to remove apoptotic bodies and debris), and then filtered (0.22 µm). The filtrate was subjected to ultracentrifugation at 100 000 × g for 70 min. The pellet was resuspended in PBS, pooled, and purified by a second round of ultracentrifugation. The final exosome pellet was resuspended in 500 µL PBS.^[^ ^69^ ^]^ Isolated exosomes were characterized by particle size analysis, electron microscopy, and surface marker profiling as previously described.^[^ ^70^, ^71^ ^]^

### Quantitation of Cellular Acetyl‐CoA

Cellular acetyl‐CoA was analyzed with the acetyl‐CoA assay kit (Abcam) according to the manufacturer's instructions. The fluorescent signal was measured at excitation/emission wavelengths of 535/589 nm.^[^ ^72^ ^]^ The PicoProbe assay was performed in 96‐well black plates, and the resulting fluorescence was quantified using the Synergy H4 Hybrid Reader (BioTek Instruments).

### In Vitro Ubiquitination Assay

For the in vitro ubiquitination assay, HA‐LIS1 and Flag‐Blimp1 were individually immunoprecipitated from transfected 293T cell lysates using anti‐HA and anti‐Flag magnetic beads, respectively. The proteins were then eluted from the beads using their corresponding HA (Sigma–Aldrich, I2149) or Flag (Sigma–Aldrich, F4799) peptides. The eluted LIS1 and Blimp1 proteins were incubated with E1 enzyme, E2 enzyme, ubiquitin, DTT, and reaction buffer at 37 °C for 6 h using a commercial ubiquitination kit (Enzo Life Sciences, #BML‐UW9920). The ubiquitination levels of Blimp1 were subsequently analyzed by immunoblotting.^[^ ^73^ ^]^

### ^13^C Metabolic Flux Analysis

CSCs were pre‐cultured in medium containing [13C_2_]‐sodium acetate (Sigma–Aldrich, 282014) and then switched to standard medium prior to co‐culture with CD8⁺ T cells. After co‐culture, CD8⁺ T cells were isolated by immunomagnetic sorting, counted, and subjected to 13C metabolic flux analysis. Data were normalized to cell number. For metabolite extraction, cells were washed with PBS, pelleted, and extracted in ice‐cold 80% methanol. The extracts were centrifuged, and the supernatants were collected, dried under a nitrogen stream, and stored at −80 °C until analysis. Metabolites were separated on a Waters ACQUITY HSS T3 column and analyzed by UPLC‐MS (Agilent ESI) in both positive and negative modes. Data were processed using EI‐MAVEN, and 13C enrichment was corrected for natural isotope abundance using the AccuCor package. CD8⁺ T cells co‐cultured with unlabeled CSCs served as the blank control. All conditions included three biological replicates.

### BMS‐303141@aCD133 and DAPT@aCD133 Nanoparticles

Bovine serum albumin (BSA) was reacted with N‐Hydroxysuccinimide (NHS)‐functionalized FAM overnight at a molar ratio 1:3 in PBS, followed by purification with ultrafiltration (MWCO = 10 kDa) to obtain BSA‐FAM. BSA‐FAM (in DI water) and drugs (in DMSO) were mixed at different mass ratios (BSA: BMS‐303141 = 10:1, BSA: DAPT = 20:1) and the final solution was adjusted so that volume ratio of deionized (DI) water to DMSO is 5:1. Then, bifunctional cross‐linker NHS‐SS‐NHS was added to the mixture (molar ratio of BSA: linker = 1:25) for reaction at room temperature on shaker. After reaction for 6 h, DI water was added to lower the concentration of DMSO, and then CD133 antibody was added to the mixture (mass ratio of BSA:antibody = 20:1) for another 3 h of reaction. The reaction mixture was then dialyzed in a dialysis bag (MWCO = 300 kDa) for 24 h before centrifugation at 2000 rpm for 5 min. The supernatant was harvested and freeze‐dried for future use. A small portion of the dried sample was weighed and dissolved in methanol for absorbance measurement (at 254 nm) via UV–vis spectroscopy to quantify the drug loading amount based on standard curves of drugs.

### Antibody‐Drug Conjugate (ADC) Preparation

Anti‐mouse CD103 antibody (M290) and maleimidocaproyl monomethylauristatin F (MC‐MMAF) were obtained from MedChemExpress (MCE). To deplete CD103‐expressing T cells, the M290‐MC‐MMAF conjugate was synthesized and applied as previously described.^[^ ^74^, ^75^ ^]^

### DC Maturation and Macrophage Polarization

Human monocytes were isolated from PBMCs using a human monocyte enrichment kit (STEMCELL Technologies, 19059). For M1 macrophage polarization, monocytes were differentiated with M‐CSF for 7 days (100 ng mL^−1^, Novoprotein), followed by stimulation with LPS (100 ng mL^−1^, Sigma–Aldrich) and IFN‐γ (20 ng mL^−1^, Novoprotein) for 24 h. For M2 macrophage polarization, monocytes were pretreated with M‐CSF (100 ng mL^−1^, Novoprotein) for 7 days, then polarized with IL‐4 (20 ng mL^−1^, Novoprotein) and IL‐10 (20 ng mL^−1^, Novoprotein) for 24 h. For dendritic cell differentiation, monocytes were cultured with GM‐CSF (70 ng mL^−1^, Novoprotein) for 7 days and subsequently treated with IL‐4 (50 ng mL^−1^, Novoprotein) for 24h.^[^ ^76^ ^]^

### Statistics

Data were presented as mean ± SEM. Two‐tailed Student's *t*‐test for comparisons between two groups, one‐way ANOVA with Tukey's method for multi‐group comparisons, two‐way ANOVA for multifactorial analyses, and Pearson correlation coefficient for assessing linear relationships. All statistical analyses were performed using GraphPad Prism 10.2.3, with a *p* < 0.05 considered statistically significant.

## Conflict of Interest

The authors declare no conflict of interest.

## Author Contributions

J.L., H.J., and J.G. contributed equally to this work. Z.W. designed and supervised the study. J.L., H.J., J.G., M.L., D.S., and M.Z. performed experiments and analyzed data. T.R. and L.X. collected samples and contributed to data analyses. J.G., T.R., Q.C., and Y.Z. contributed to the study design and provided critical materials. J.L. and Z.W. wrote the manuscript with input from all authors.

## Supporting information



Supporting Information

## Data Availability

The data that support the findings of this study are available from the corresponding author upon reasonable request.
