# Supplementary material for: Cancer Stem Cells Shift Metabolite Acetyl‐Coenzyme A to Abrogate the Differentiation of CD103+ T Cells
Source: Adv Sci (Weinh). 2025 Nov 23;13(8):e13535. doi: 10.1002/advs.202513535 (PMC12884792; doi:10.1002/advs.202513535)
Supplement: Supplementary file 1 — Supporting Information [file ADVS-13-e13535-s001.pdf]

Supplementary Figure 1

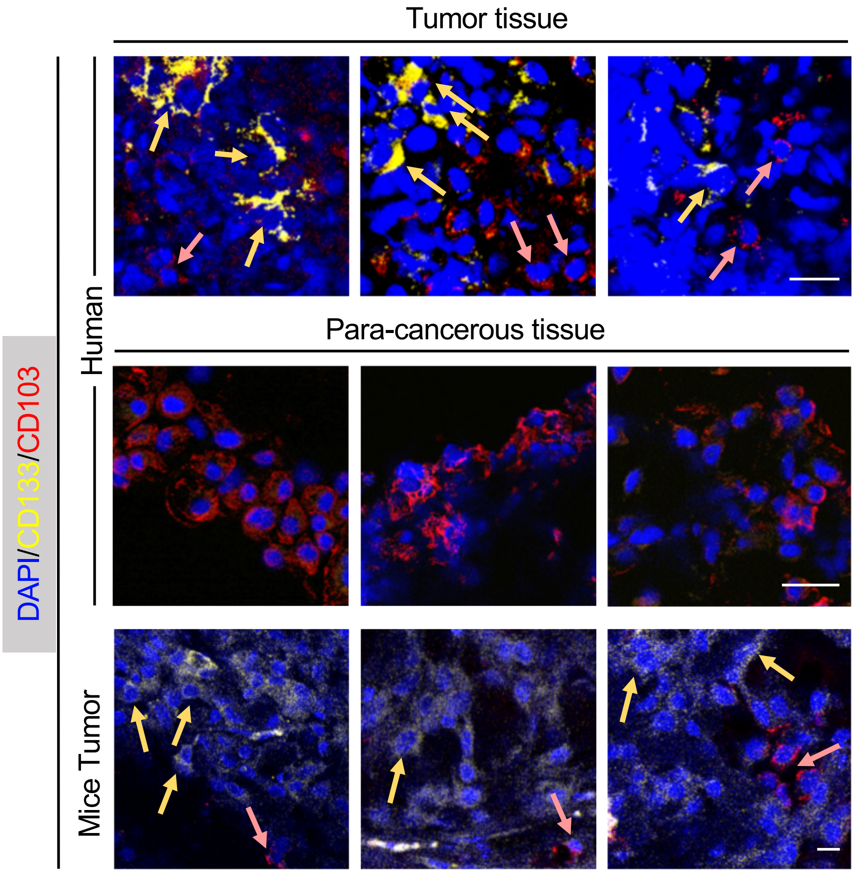

Fig. S1 Co-localizations of CD133⁺ CSCs and CD103⁺ T cells in NSCLC TME

Immunofluorescence staining of $CD103^+$ T cells (red) and $CD133^+$ tumor cells (yellow) in human NSCLC sections. Representative images are shown for patient-derived lung tumor tissue, para-tumor tissue, and tumor tissue from a humanized NSCLC chimera model. Nuclei are counterstained with DAPI (blue). Co-localization of $CD103^+$ T cells and $CD133^+$ tumor cells is observed in the tumor microenvironment. Scale bar: 20 μm.

Supplementary Figure 2

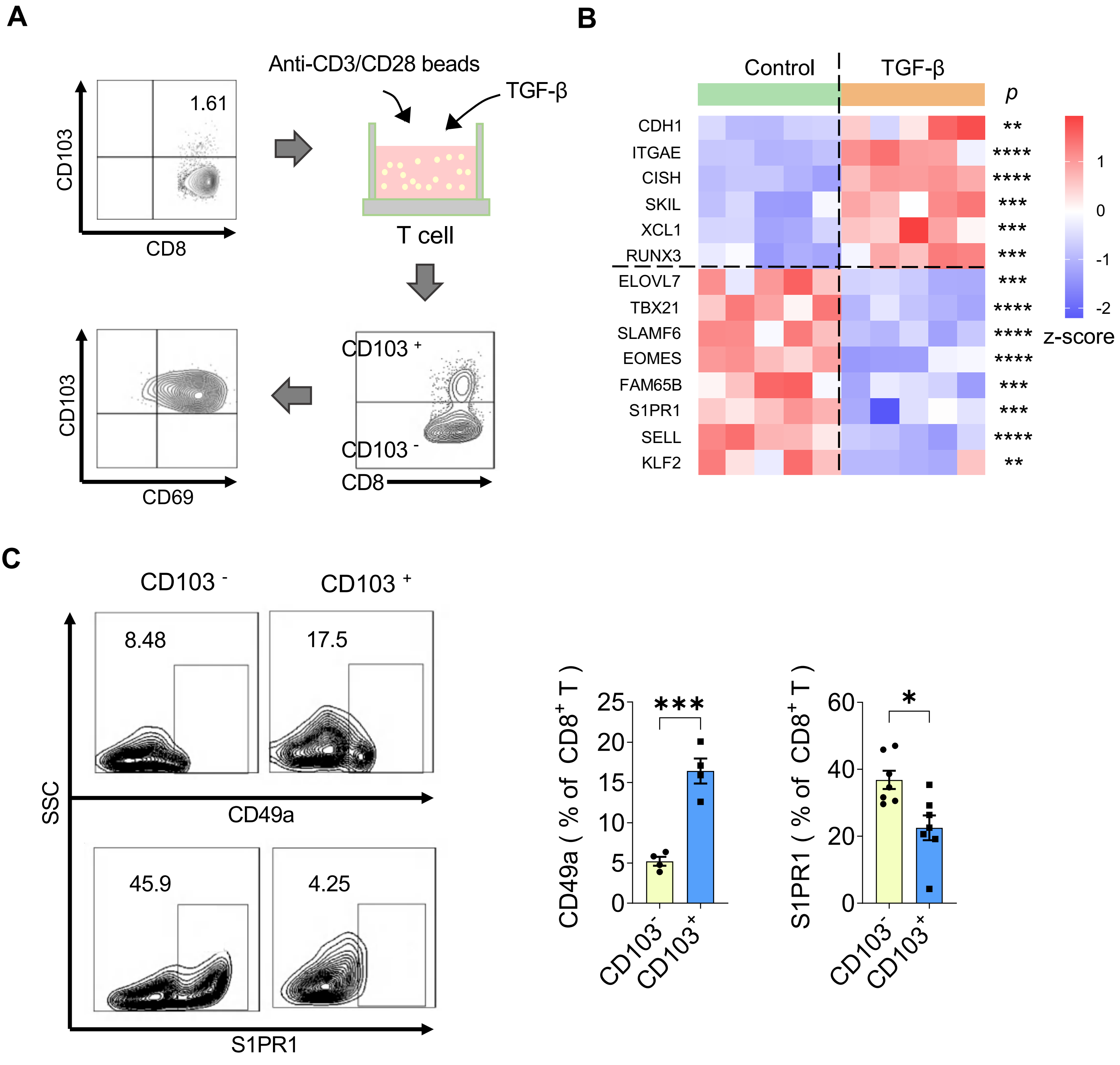

Fig. S2 TGF-β induces the formation of CD103⁺ T cells

(A) Schematic diagram for experimental design. (B) CD8⁺ T cells from healthy individuals were activated for 72h in the presence or absence of TGF-β (10 ng/mL). CD103⁺ T cells-related transcripts were determined by qPCR. Shown from 5 independent experiments. (C) Expression of CD49a and S1PR1 was analyzed within gated CD103⁺ and CD103⁻ T cells. One representative dot blot from 4-7 healthy donors. *$p < 0.05$, **$p < 0.01$, ***$p < 0.001$ and ****$p < 0.0001$ with paired t test (B-C).

Supplementary Figure 3

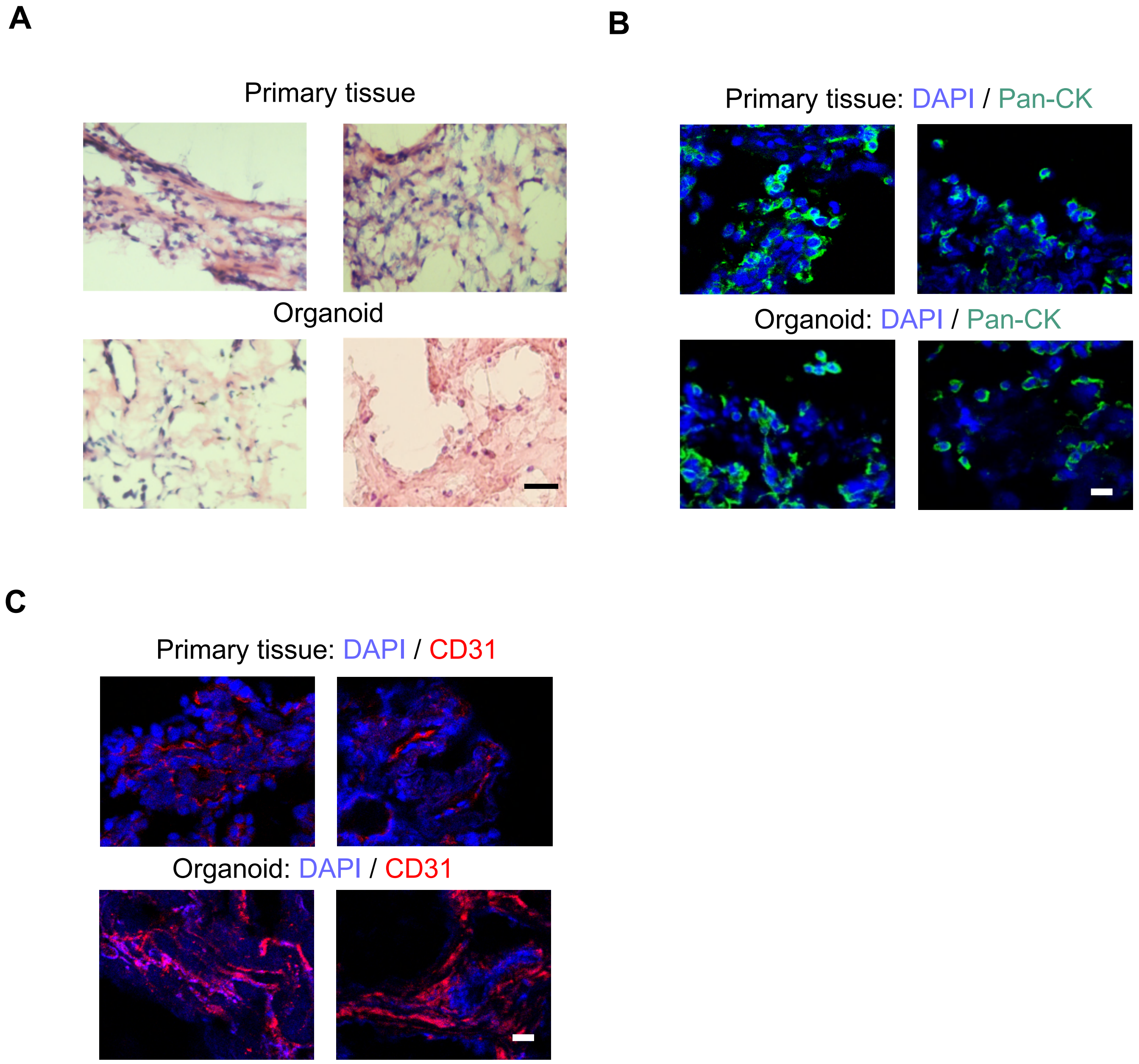

Fig. S3 Establishment of PDOs

(A) Representative HE staining images of 2 PDO-primary tumor pairs. Scale bar, 100 μm. (B) Representative immunofluorescence images of 2 PDO-primary tumor pairs with immunostaining for Pan-Ck. Nuclei were stained with Hoechst. Scale bar, 20 μm. (C) Representative immunofluorescence images of 2 PDO-primary tumor pairs with immunostaining for CD31. Nuclei were stained with Hoechst. Scale bar, 20 μm.

Supplementary Figure 4

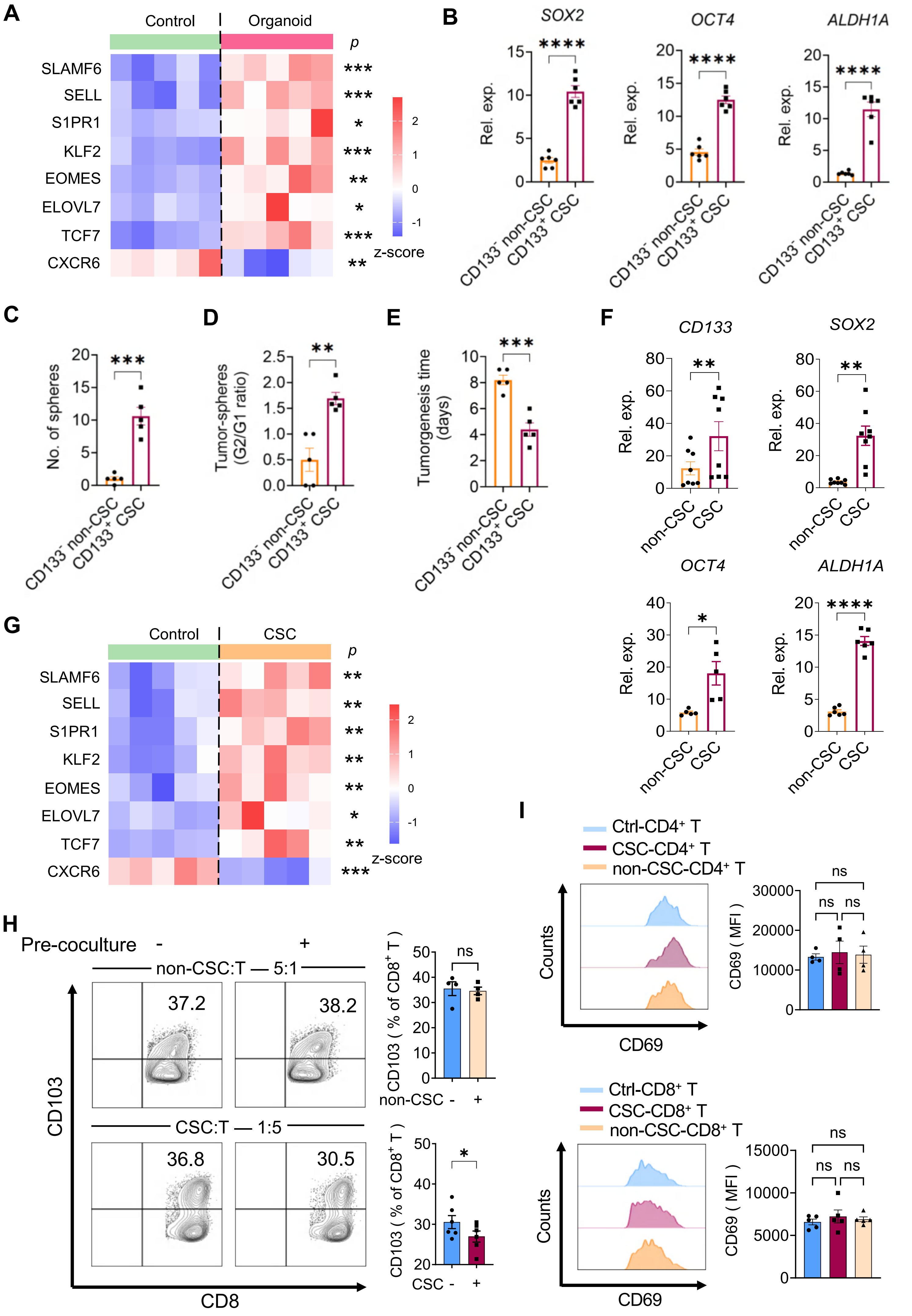

Supplementary Figure 4

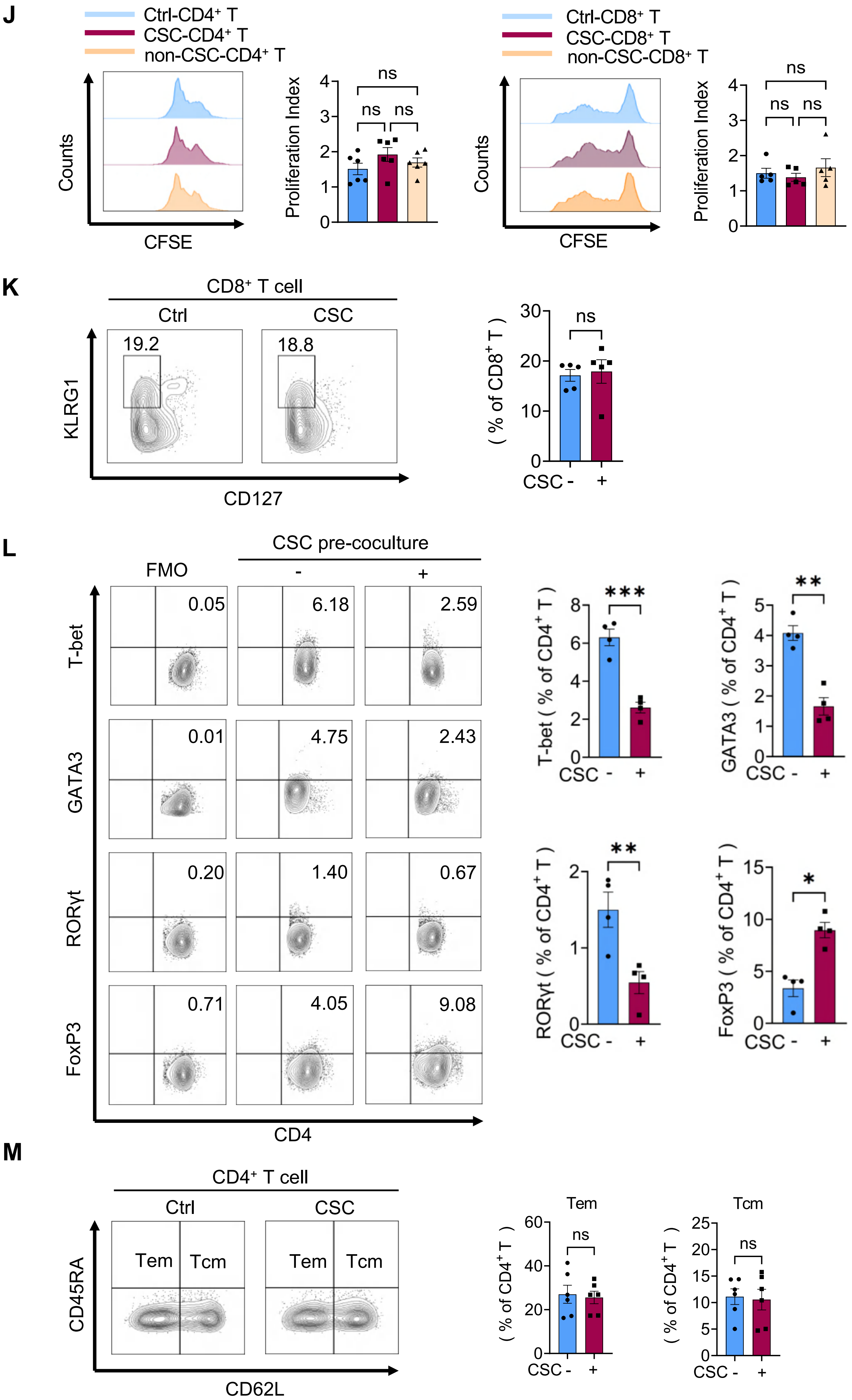

Supplementary Figure 4

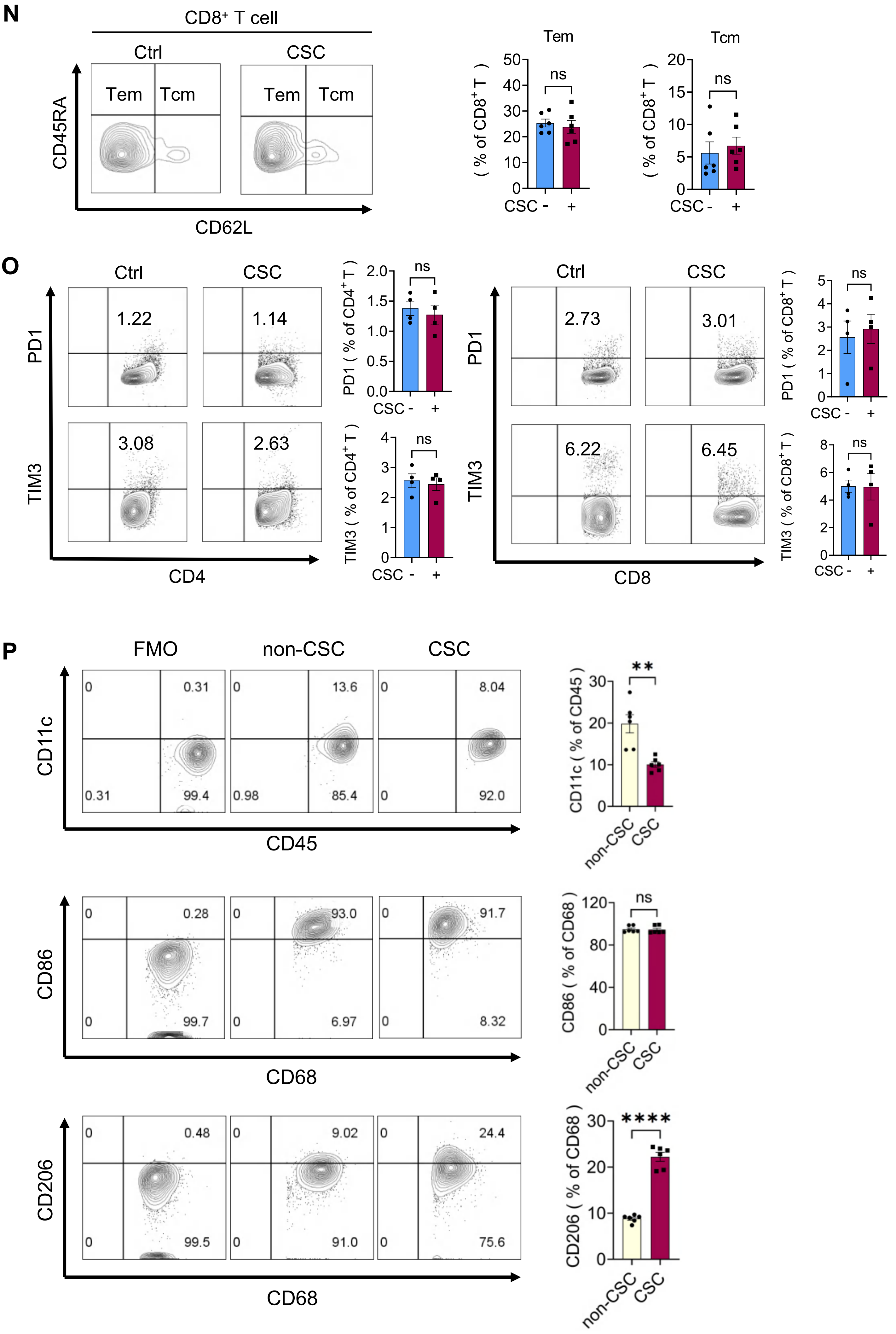

Supplementary Figure 4

Fig. S4 NSCLC-T cell interactions

(A) $CD8^+$ T cells were pre-conditioned with PDOs followed by induction of $CD103^+$ T cells as indicated. mRNA of $CD103^+$ T cells-related transcripts was tested by qPCR. Mean ± SEM from 5 donors. (B) qRT-PCR analysis of stemness-associated gene expression in FACS-sorted $CD133^+$ and $CD133^-$ tumor cells. Mean ± SEM from 6 patients. (C) Tumor sphere-forming capacity of $CD133^+$ and $CD133^-$ cells was assessed in vitro. Mean ± SEM from 5 patients. (D) Quantification of self-renewal potential, presented as tumor sphere formation efficiency at serial passages (G1 and G2). Mean ± SEM from 5 patients. (E) In vivo tumor initiation capacity was tested by transplantation of $CD133^+$ and $CD133^-$ tumor cells into NSG mice. Mean ± SEM from 5 patients. (F) The expression of CD133, SOX2, OCT4, and ALDH1 in CSCs and non-CSCs was detected by qPCR. Mean ± SEM from 5-8 independent experiments. (G) Healthy $CD8^+$ T cells were co-cultured with CSCs for 24 hours, followed by induction of $CD103^+$ T cells ex vivo. qPCR was employed to detect the $CD103^+$ T cell-related transcripts. Mean ± SEM from 5 donors. (H) Healthy $CD8^+$ T cells were pre-conditioned with CSCs and non-CSCs (at a ratio of 1:5 and 5:1) for 24h. $CD8^+CD103^+$ T cells were induced with anti-CD3/CD28 beads plus TGF-β (10 ng/mL) for 72h. $CD103^+$ T cells were tested by flow cytometry. Mean ± SEM from 4-6 donors. (I) After CSCs and non-CSCs pretreatment, CD69 expression on T cells was detected upon TCR stimulation for 12h by FACS. Mean ± SEM from 4-5 donors. (J) T cells were pre-conditioned with CSCs and non-CSCs for 24h, followed by proliferation analysis of T cells upon stimulation with anti-CD3/CD28 beads for 72h. Mean ± SEM from 5-6 donors. (K-O) T cells were co-cultured with CSC for 24h, then the proportion of Teff (K), Th1, Th2, Th17, Treg (L), Tem, Tcm (M-N) and Tex (O) were measured in the presence of anti-CD3/CD28 beads as a stimulator for 72h (K, M-N, O) or 96h (L). Mean ± SEM from 4-6 donors. (P) $CD14^+$ cells were isolated from PBMCs and pre-educated by CSCs or non-CSCs for 24h. Then DCs were induced with GM-CSF (70 ng/mL) for 7d and IL-4 (50 ng/mL) for 24h. M1 macrophages were induced with M-CSF (100 ng/mL) for 7d, LPS (100 ng/mL) and IFN-γ (20 ng/mL) for 24h. M2 macrophages were induced with M-CSF (100 ng/mL) for 7d, IL-4 (20 ng/mL) and IL-10 (20 ng/mL) for 24h. Mean ± SEM from 6 donors. *$p < 0.05$, **$p < 0.01$, ***$p < 0.001$ and ****$p < 0.0001$ with unpaired (F) and paired (A-E, G-H, L, P) t-test.

Supplementary Figure 5

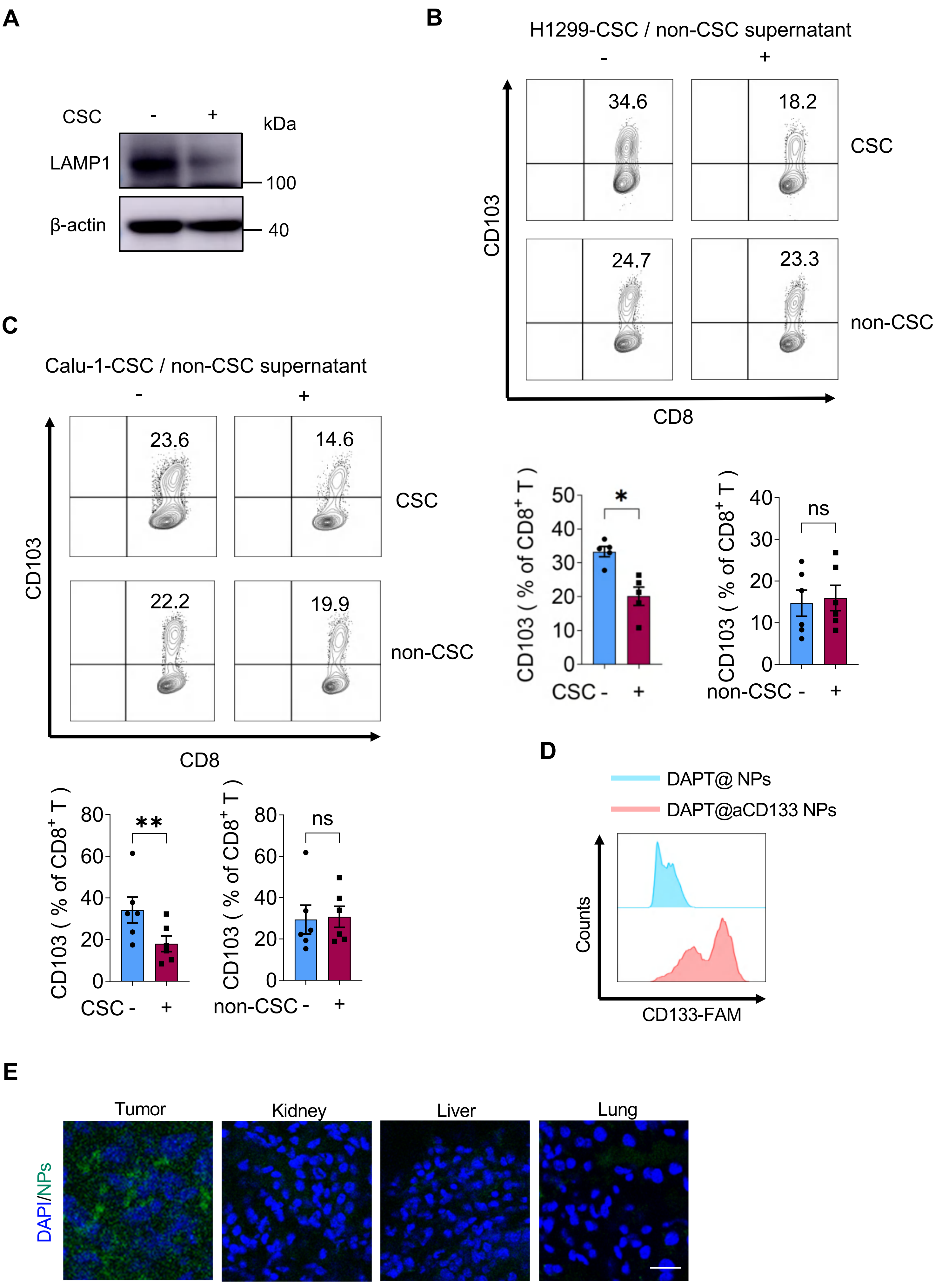

Supplementary Figure 5

F

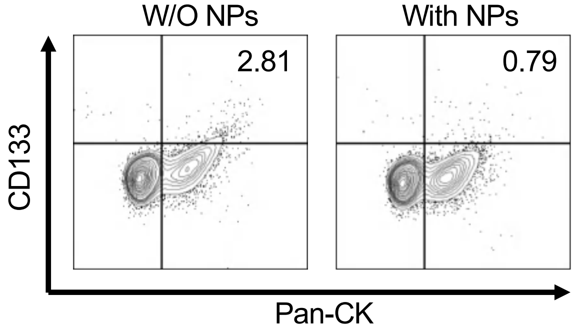

G

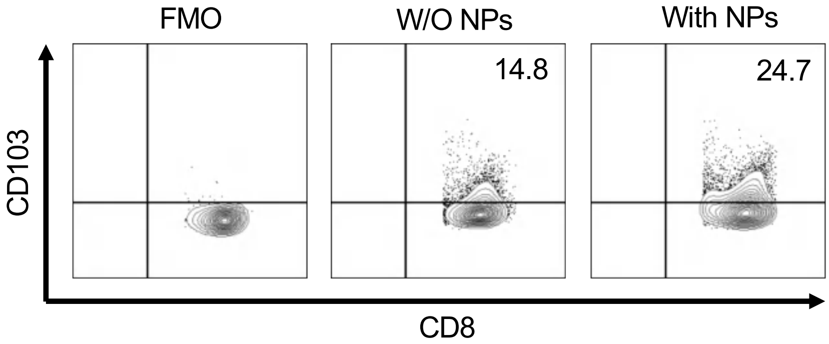

H

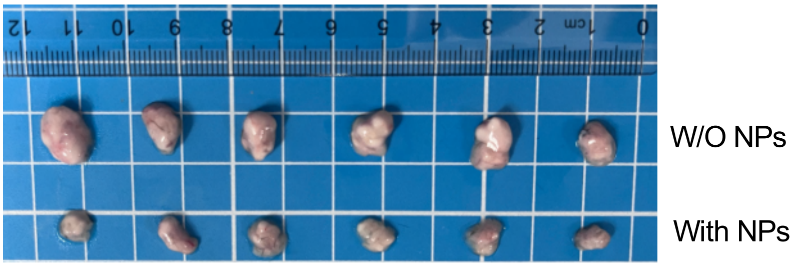

Fig. S5 CSCs inhibit CD103^{+} T cells differentiation

(A) CD8^{+} T cells were pre-conditioned with CSCs for 24h, followed by the induction of CD103^{+} T cells. LAMP1 was detected in induced T cells by immunoblot. (B-C) CD8^{+} T cells were pre-conditioned with non-CSCs and CSCs (Calu-1 and Calu-1 tumor spheres, H1299 and H1299 tumor spheres), followed by the induction of CD103^{+} T cells ex vivo. The percentage of CD8^{+}CD103^{+} T cells was detected with flow cytometry. Mean ± SEM from 5-6 individuals in each group. (D) Shown are sample histograms of CSCs incubated with or without DAPT@aCD133 NPs. (E) Representative fluorescence images showing the biodistribution and accumulation of NPs in major organs and tumors at 24 h post-injection. (F) Representative flow cytometry plots analyzing the frequency of CD133^{+} cells within tumors from different treatment groups. (G) Representative flow cytometry plots gated on CD8^{+} T cells, showing the proportion of CD103^{+} cells in the TME post-treatment. (H) Representative gross photographs of resected tumors from mice receiving the indicated treatments. $*p < 0.05$ and $**p < 0.01$ with paired t-test (B-C).

Supplementary Figure 6

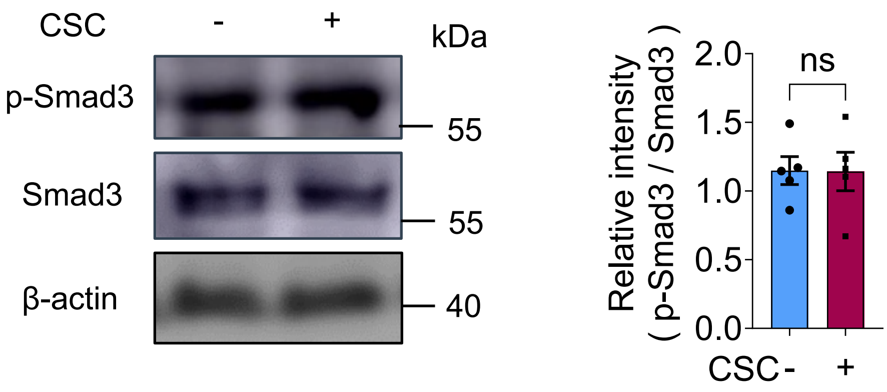

Fig. S6 CSCs exert no effect on the TGF-β-Smad3 signaling in T cells

Healthy $CD8^+$ T cells were co-cultured with CSCs for 24h followed by induction of $CD103^+$ T cells ex vivo. The protein levels of Smad3 and p-Smad3 were analyzed by immunoblot. Mean ± SEM from 5 individuals in each group.

Supplementary Figure 7

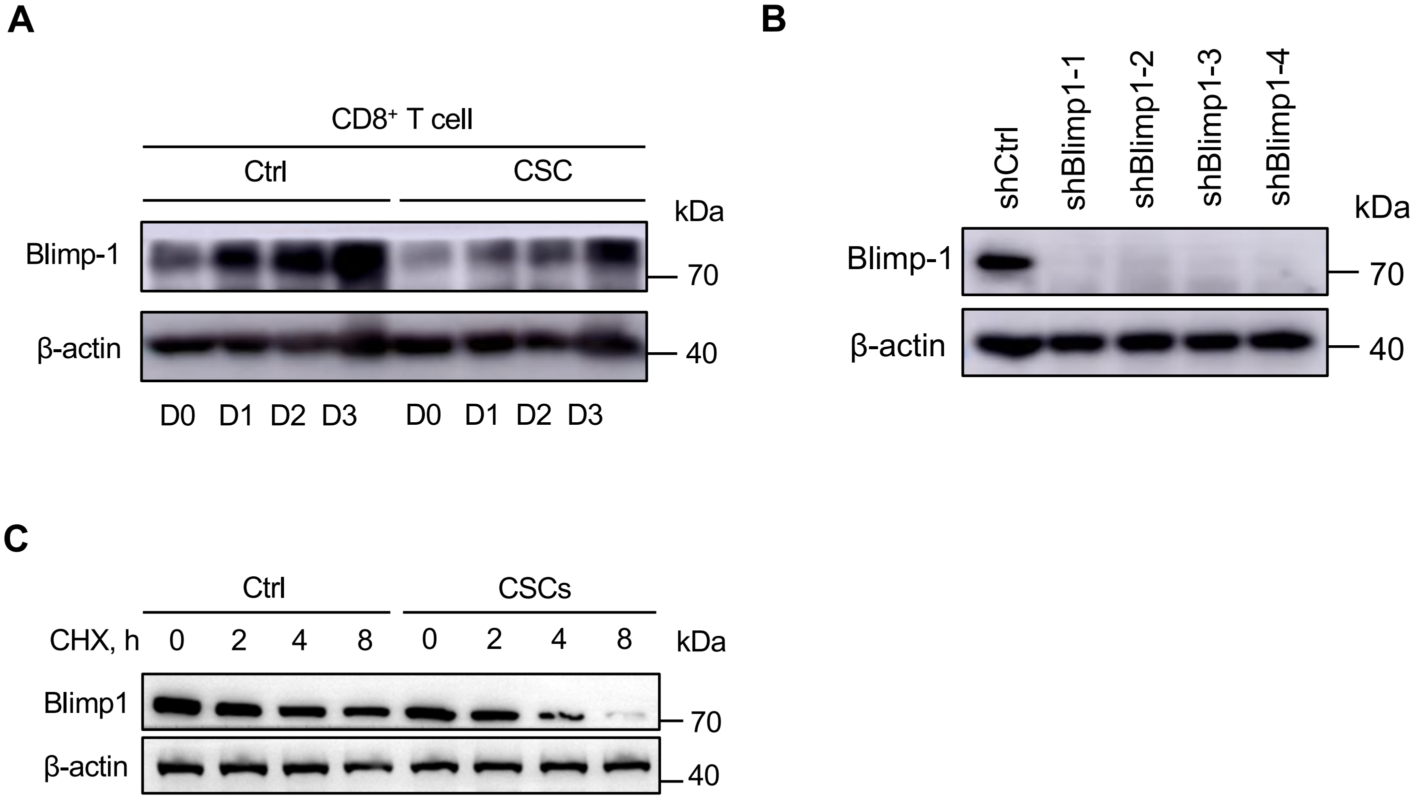

Fig. S7 Blimp-1 is the target of CSC on T cells

(A) Healthy $CD8^+$ T cells were co-cultured with CSCs for 24h, followed by induction of $CD103^+$ T cells ex vivo. Immunoblotting was performed to detect Blimp-1 in the indicated $CD8^+$ T cells. (B) Genetic knockdown efficiency of Blimp-1 in healthy T cells was assessed after lentiviral shRNA transfections. (C) $CD8^+$ T cells pretreated with or without CSCs for 24 hours, were subjected to CHX chase assay to monitor Blimp-1 degradation.

Supplementary Figure 8

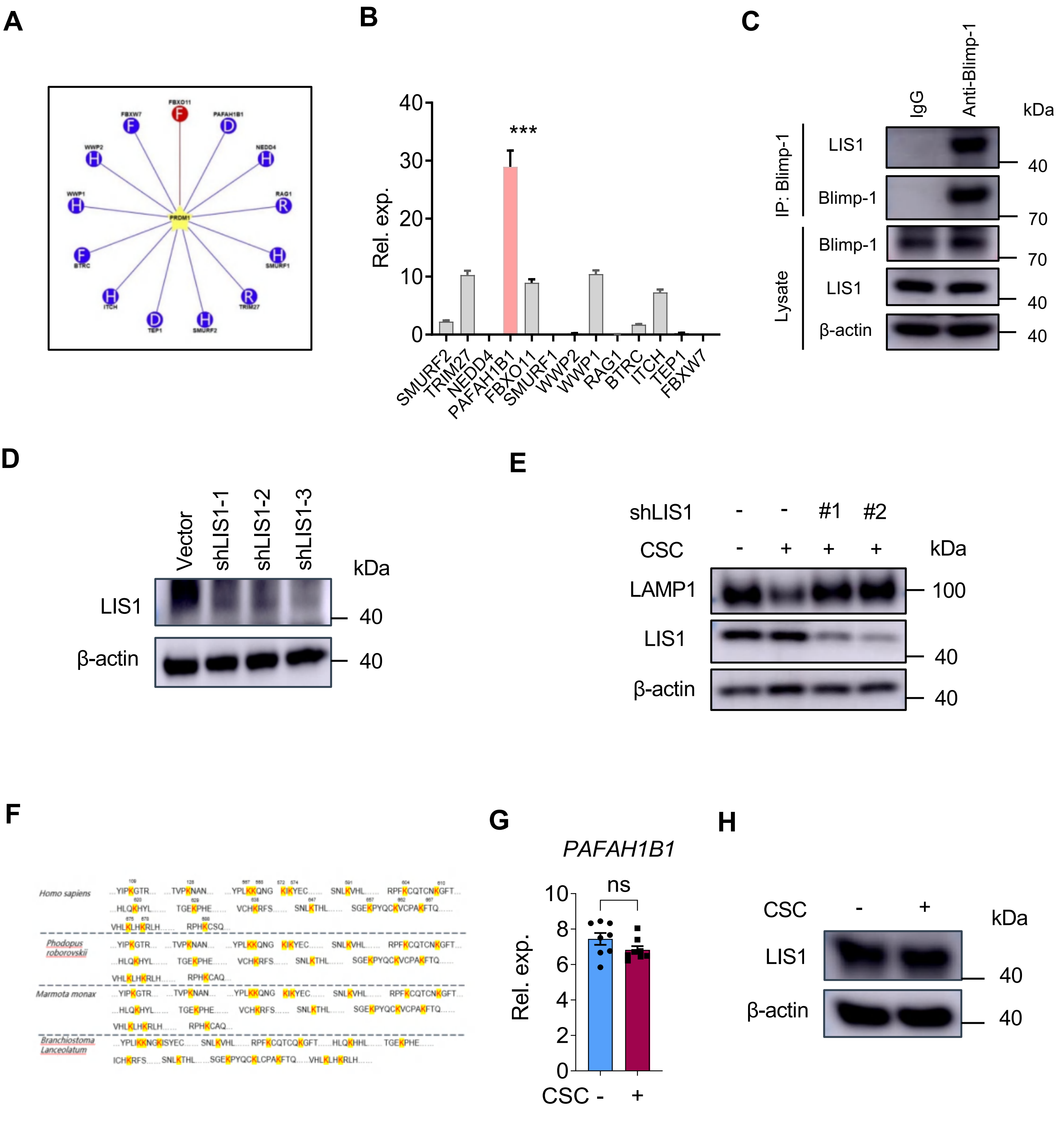

Fig. S8 LIS1 is the critical E3 ligase to Blimp-1 in CSC-T cell interaction

(A) Potential E3 ligase of Blimp-1 obtained from the UbiBrowser database. (B) qPCR analysis was performed to detect the mRNA levels of the E3 ligase in induced T cells. Mean ± SEM from 7 individuals in each group. (C) Co-immunoprecipitation analysis of endogenous Blimp-1-LIS1 interaction in induced CD8^{+}CD103^{+} T cells. (D) Immunoblotting was performed to assess LIS1 levels in HEK293T cells transfected with three specific shRNAs targeting LIS1. (E) CD8^{+} T cells were co-cultured with CSCs for 24h, either with or without LIS1-targeting shRNA transfection, followed by induction of CD103^{+} T cells. Blimp-1 expression in the resulting CD8^{+} T cell populations was analyzed by immunoblotting. (F) Alignment of Blimp-1 amino acid sequence from various species. Yellow shading indicates the conserved lysines. (G-H) qPCR and western blot analysis of LIS1 (PAFAH1B1) in induced T cells. Mean ± SEM from 8 individuals in each group. ***$p < 0.001$ with ANOVA and Tukey's method (B).

Supplementary Figure 9

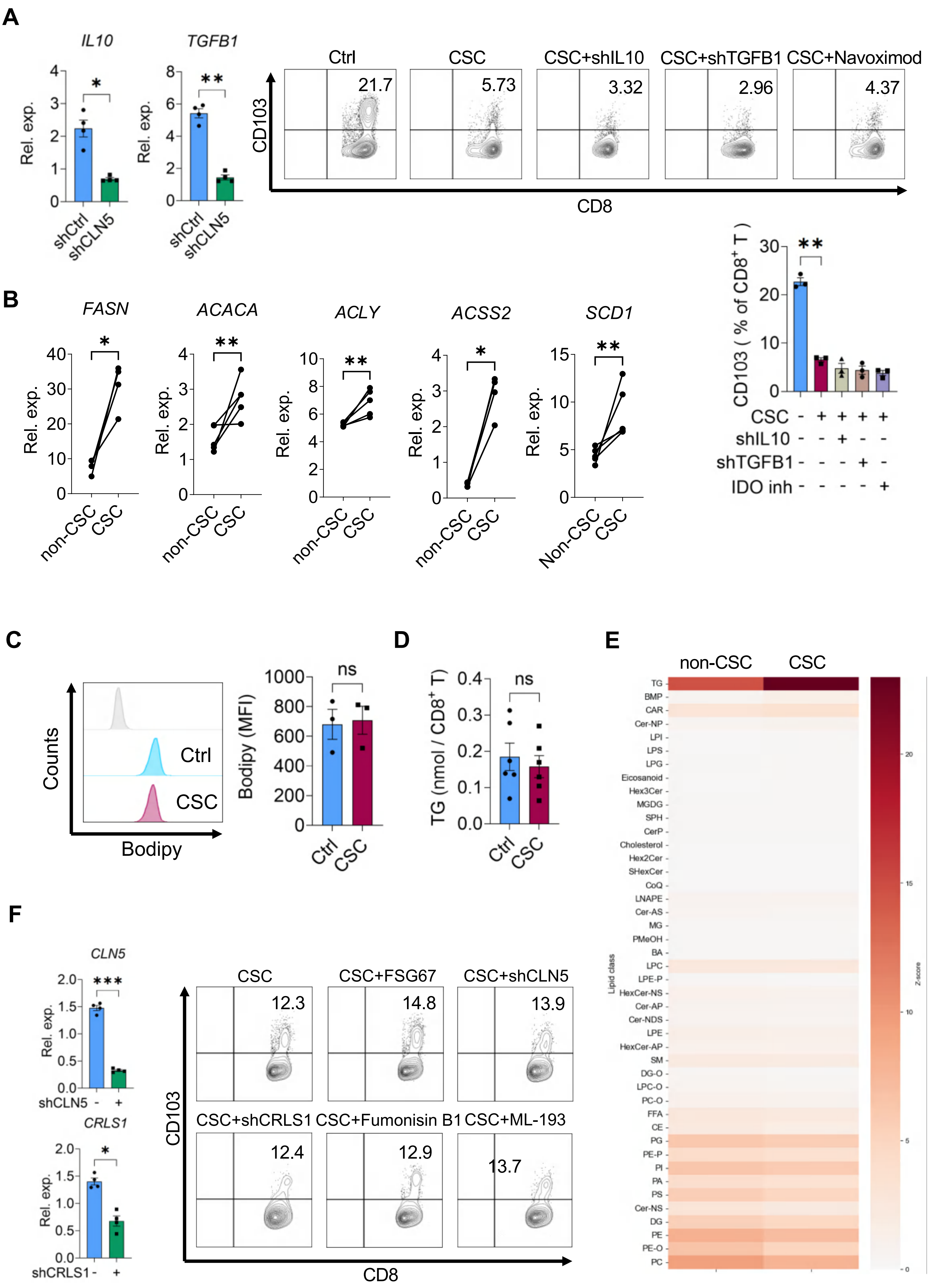

Supplementary Figure 9

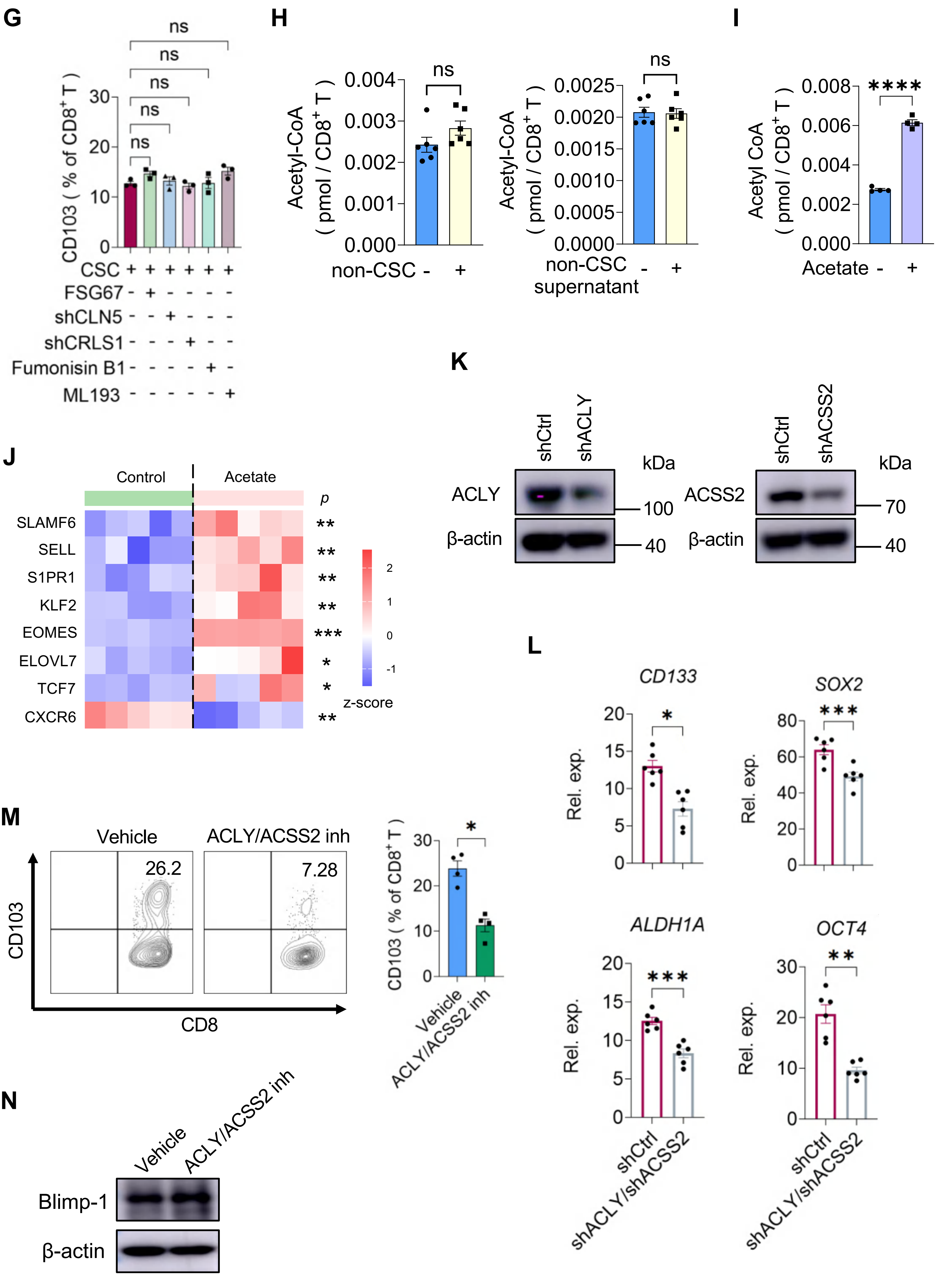

Fig. S9 CSC-derived acetyl-CoA, but not other metabolites, inhibits CD103⁺ T cell differentiation

(A) Cancer stem cells (CSCs) were transfected with shIL10, shTGFB1, or pretreated with the IDO inhibitor Navoximod (100 nM) for 24 h, followed by coculture with healthy $CD8^+$ T cells for an additional 24 h. The percentage of ex vivo-induced $CD8^+CD103^+$ T cells was quantified by flow cytometry. Data are presented as mean ± SEM from 3 individuals per group. (B) Expression of lipogenesis-related genes in CSCs versus non-CSCs was determined by qPCR. Results are from 5 independent experiments. (C) Bodipy staining in $CD8^+$ T cells with or without CSC pretreatment was analyzed by flow cytometry. Data are shown as mean ± SEM from 3 individuals per group. (D) TG-associated Bodipy levels in $CD8^+$ T cells (with or without CSC pretreatment) were measured using an assay kit. Data are shown as mean ± SEM from 6 individuals per group. (E) Metabolomic analysis identified the metabolite with the largest variation range between CSCs and non-CSCs. (F-G) CSCs were transfected with shCLN5, shCRLS1, or pretreated with the TG inhibitor FSG67 (50 μM) or the ceramide nanoparticle (Cer-NP) inhibitor Fumonisin B1 (10 mM) for 24 h, then cocultured with healthy $CD8^+$ T cells for an additional 24 h. Alternatively, $CD8^+$ T cells were treated with a GPR55 inhibitor (targeting the lysophosphatidylinositol [LPI] receptor) (10 mM) during the 24-h coculture with CSCs. The percentage of ex vivo-induced $CD8^+CD103^+$ T cells was quantified by flow cytometry. Data are presented as mean ± SEM from 3 individuals per group. (H) Acetyl-CoA levels in CD8+ T cells after incubating with non-CSCs and non-CSCs supernatant for 24h. Mean ± SEM from 6 individuals in each group. (I) Acetyl-CoA in CD8+ T cells treated ± acetate (10 mM) for 24h. Mean ± SEM from 4 individuals in each group. (J) qPCR analysis of CD103+ T cells-associated transcripts in induced CD8+CD103+ T cells in the presence or absence of acetate (10 mM). Mean ± SEM from 5 individuals in each group. (K) Genetic knockdown efficiency of ACLY and ACSS2 in CSCs by lentiviral shRNA transfections. (L) The expression of CD133, SOX2, OCT4, and ALDH1 in CSCs transfected with shACLY or shACSS2 versus controls. Shown from 6 independent experiments. (M-N) $CD8^+$ T cells were activated and induced for 3 days in the presence of ACLY and ACSS2 inhibitors. The frequency of $CD8^+CD103^+$ T cells was determined by flow cytometry, and Blimp-1 expression was analyzed by western blot. Mean ± SEM from 4 individuals. *$p < 0.05$, **$p < 0.01$, ***$p < 0.001$ and ****$p < 0.0001$ with unpaired (B), paired (I, J ,M) t-test and ANOVA plus Tukey's method (A, F).

Supplementary Figure 10

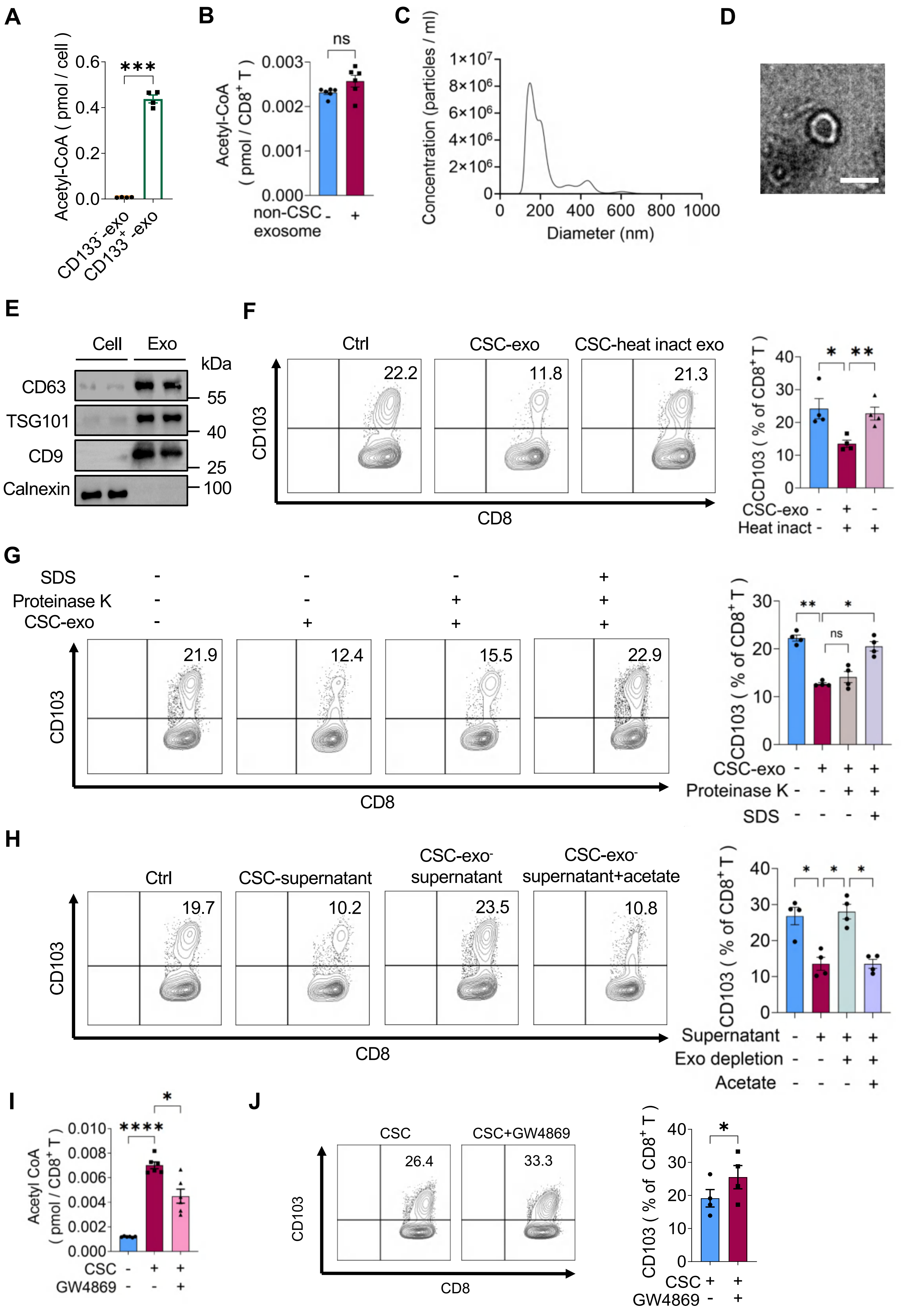

Supplementary Figure 10

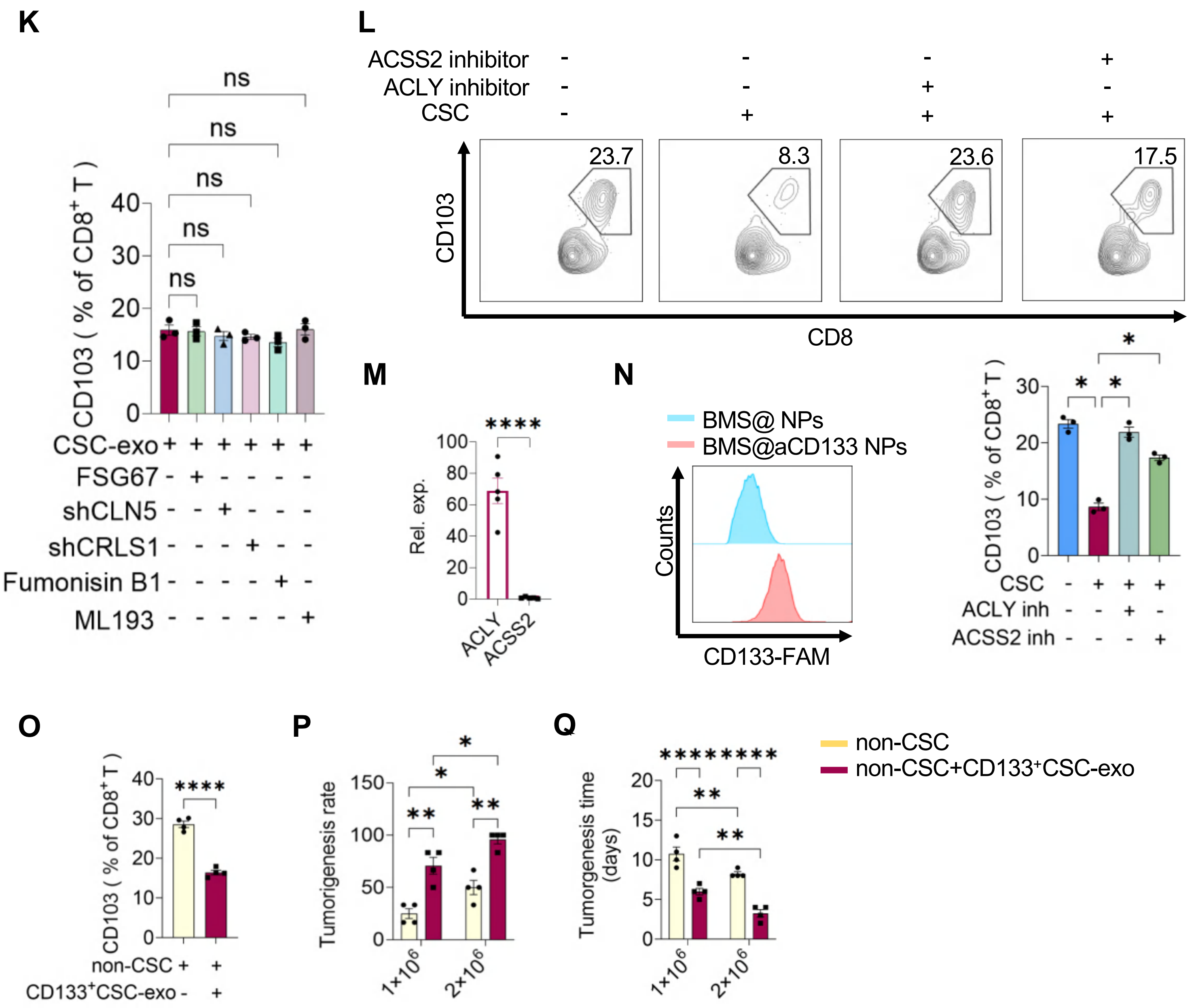

Fig. S10 non-CSCs could not increase the intracellular level of acetyl-CoA in interacted T cells

(A) Acetyl-CoA levels were measured in exosomes derived from PDO-derived primary $CD133^+$ and $CD133^-$ tumor cells. Data are presented as mean ± SEM from 4 individuals per group. (B) Acetyl-CoA levels were determined in $CD8^+$ T cells following 24-h coculture with exosomes isolated from non-CSCs. Data are shown as mean ± SEM from 6 individuals per group. (C) NTA was performed to characterize exosomes isolated from the conditioned medium of CSCs. (D) Representative TEM image of the isolated exosomes. Scale bar: 100 nm. (E) Exosome markers were detected by western blot. (F-G) $CD8^+$ T cells were co-cultured for 24 hours with CSC-derived exosomes that were either left untreated, heat-inactivated, or treated with Proteinase K in the presence or absence of SDS. Following exosome exposure, T cells were activated and induced to differentiate. The frequency of $CD8^+CD103^+$ T cells was quantified by flow cytometry. Data are shown as mean ± SEM from 4 individuals. (H) $CD8^+$ T cells were co-cultured for 24 hours with CSC-conditioned medium that was either exosome-depleted or left untreated. The T cells were then induced to differentiate ex vivo into $CD8^+CD103^+$ T cells in the presence or absence of acetate (10 mM). The resulting frequency of $CD8^+CD103^+$ T cells was quantified by flow cytometry. Mean ± SEM from 4 individuals in each group. (I-J) CSCs were pretreated with the exosome inhibitor GW4869 (10 μM) for 24h before coculturing with healthy $CD8^+$ T cells for an additional 24h. Acetyl-CoA levels in $CD8^+$ T cells were then measured, followed by induction of $CD8^+CD103^+$ T cell differentiation as described. Mean ± SEM from 4-6 individuals in each group. (K) CSCs were perturbed for 24 h with lipid-pathway-targeting shRNAs (shCLN5, shCRLS1) or inhibitors (FSG67 for TG, Fumonisin B1 for Cer-NP). Exosomes were subsequently isolated from the conditioned media of these pretreated CSCs and added to $CD8^+$ T cell cultures for 24 h. Separately, $CD8^+$ T cells were co-cultured with untreated CSCs in the presence of a GPR55 antagonist. The frequency of $CD8^+CD103^+$ T cells following ex vivo induction was analyzed by flow cytometryData are presented as mean ± SEM from 3 individuals per group. (L) CSCs were pretreated with the ACLY inhibitor BMS303141 or the ACSS2 inhibitor, and then co-cultured with $CD8^+$ T cells for 24 h. The induced $CD8^+CD103^+$ T cells were analyzed by flow cytometry. (M) qPCR analysis was performed to detect the mRNA levels of the ACLY and ACSS2 in CSCs. Mean ± SEM from 5 independent experiments. (N) Shown are sample histograms of CSCs incubated with or without BMS@aCD133 NPs. (O) PDO-derived non-CSCs (2×10^6) mixed with CSC-derived exosomes (10 μg) were co-implanted subcutaneously into NSG mice. The proportion of intratumoral $CD8^+CD103^+$ T cells was quantified by flow cytometry. (P-Q) PDO-derived non-CSCs (1×10^6 or 2×10^6 cells) mixed with CSC-derived exosomes (10 μg) were co-implanted subcutaneously into NSG mice. Tumor incidence and time to tumor onset were recorded. *$p < 0.05$, **$p < 0.01$, ***$p < 0.001$ and ****$p < 0.0001$ with unpaired (M), paired t test (A, J, O-Q) and ANOVA plus Tukey's method (F-I, L).

Supplementary Figure 11

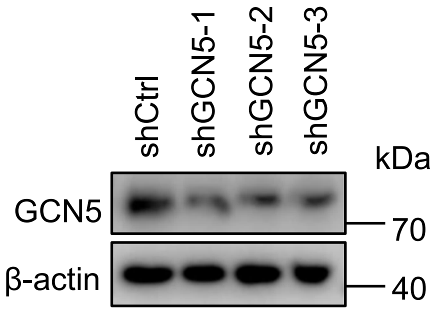

Fig. S11 Genetic knockdown of GCN5 in T cells

GCN5 knockdown efficiency in healthy T cells achieved by lentiviral shRNA transduction.

Supplementary Table 1

Demographic and clinical characteristics of NSCLC patients

Demographic parameters	Patients with NSCLC	Healthy individuals
No. of subjects	91	229
Sex (F/M)	44/47	119/110
Age (mean ± SEM [years])	52.7 ± 19.3	47.2 ± 7.1
TNM staging (I/II/III)	51/36/4	N/A
Disease duration (mean ± SEM [months])	5.0 ± 3.1	N/A
Histological types		
Adenocarcinoma	72	N/A
Squamous carcinoma	19	N/A

Supplementary Table 2

Sequences of the primers for qPCR

18srRNA	F	AGTCCCTGCCCTTTGTACACA
	R	GATCCGAGGGCCTCACTAAAC
CD133	F	GCACTCTATACCAAAGCGTCAA
	R	CTCCCATACTTCTTAGTTTCCTCA
SOX2	F	TACAGCATGTCCTACTCGCAG
	R	GAGGAAGAGGTAACCACAGGG
OCT4	F	ACATCAAAGCTCTGCAGAAAGAAC
	R	CTGAATACCTTCCCAATAGAACC
ALDH1A	F	CGCAAGACAGGCTTTTCAGAT
	R	CCCTCTCGGAAGCATCCA
BLIMP-1	F	AAGCAACTGGATGCGCTATGT
	R	GGGATGGGCTTAATGGTGTAGAA
HOBIT	F	ACCGATGACAAGAAATTCACAGT
	R	GAAGGCGTGGAGGAGAAATGG
KLF2	F	TTCGGTCTCTTCGACGACG
	R	TGCGAACTCTTGGTGTAGGTC
CDH1	F	CGAGAGCTACACGTTCACGG
	R	GGGTGTCGAGGGAAAAATAGG
ITGAE	F	AGCCATGCAACACGTCTTAGA
	R	TCCTCGAATATGCCACCATCG
QPCT	F	TCTTCGGCAAATTGCAGAAGG
	R	CGGGTATCGCTCTATCAGCA
CISH	F	GAACTGCCCAAGCCAGTCAT
	R	GCTATGCACAGCAGATCCTCC
LITAF	F	ATGTCGGTTCCAGGACCTTAC
	R	TACGAAGGAGGATTCATGCCC
SKIL	F	ATCCCTTCAGATAGCTCCACAG
	R	AGGATGGGGCATTGAATGGAA
RGS1	F	TCTTCTCTGCTAACCCAAAGGA
	R	TGCTTTACAGGGCAAAAGATCAG
ABI3	F	ATCGCCCCAGAGAACCTACC
	R	GCTCTTTCGAGACAGGGTGC
ITGA1	F	GCTCCTCACTGTTGTTCTACG
	R	CGGGCCGCTGAAAGTCATT
XCL1	F	TGCTCTCTCACTGCATACATTG
	R	TGGTGTAGGTCTTGATTCTGCT
RGS2	F	ATACAAGAAGCTACAAGTGGCTG
	R	ACGAGGATAAGAGTTGTTCTCCA
CHN2	F	TTCCAAGCACGTTCCCAATGA
	R	CATGGGTCTCTGAGTGTTGTG
RUNX3	F	GCGAGGGAAGAGTTTCACCC
	R	TTGATGGCTCGGTGGTAGGT
CXCR6	F	TCCTGGTGAACCTACCCCTG
	R	AAACACCCATTCATGGATGCC
CD244	F	TCGTGATTCTAAGCGCACTGT
	R	CAGGTTCTTGTGACGTGGGAG
USP33	F	AAAATCCCTTGGTACTTGTCAGG
	R	TCGAAGAGTGGTAAGGTTCACA
ATP1B3	F	CCAAAATACCGTGACCAGATTCC
	R	ACGAAGTTGGATCAGACCTACTG
ELOVL7	F	GCCAAGTGACTTTCCTTCATGT
	R	GGCGACAATAACAAACTGGACA
TBX21	F	GGTTGCGGAGACATGCTGA
	R	GTAGGCGTAGGCTCCAAGG
SLAMF6	F	GAGTCCGCAAGGAACCTAGAG
	R	TCCCTGTTTGAATGAGTGACTGA
EOMES	F	GCCATGCTTAGTGACACCGA
	R	GGACTGGAGGTAGTACCGC

Supplementary Table 2

Sequences of the primers for qPCR

FAM65B	F	GAAAATTCCTCCGCTCTCAAGA
	R	TTCAAGGCCCTGTAGACTTCT
S1PR1	F	TCTGCTGGCAAATTCAAGCGA
	R	GTTGTCCCCTTCGTCTTTCTG
SELL	F	ACCCAGAGGGACTTATGGAAC
	R	GCAGAATCTTCTAGCCCTTTGC
BLIMP-1	F	AAGCAACTGGATGCGCTATGT
	R	GGGATGGGCTTAATGGTGTAGAA
HOBIT	F	ACCGATGACAAGAAATTCACAGT
	R	GAAGGCGTGGAGGAGAAATGG
FBXO11	F	ATCATGGACGTGATGTTGGTGTG
	R	CCACTGTAGGGTTAGCATAGGC
PAFAH1B1	F	GGCCATGACCACAATGTTTC
	R	CAGTAGCCAGTTTGCACTTC
NEDD4	F	CTTTATCCATTACCGACAG
	R	GGTGGCTTCATCTTCTC
RAG1	F	GCAGATGAGTCTGACCACGA
	R	CTTGAAAGTCCGGAGAATGC
SMURF1	F	GCTTCAAGGCTTTGCAAGGTT
	R	TGGGAGCCACCAACAAAAGT
TRIM27	F	TTCATCGCCGGGAGACATTA
	R	GGCTGAGGTTACTCCACCTT
SMURF2	F	GTGCTGGATTTCTCGGTTGT
	R	CCTCCTGTGCCTATTCGGT
TEP1	F	ATTTGTACCGTGTGAACAACAGC
	R	GAATCAAACCAACGACCCAAAG
ITCH	F	AGCGTAGTCAGCTTCAAGGAG
	R	AGGTGGCAATGGACCAAGAG
BTRC	F	TGGCTCATCTGACAACACTATC
	R	CGAATACAACGCACCAATTCC
WWP1	F	TTGCTGAGCTCATGGGAAGT
	R	TGGTGGTAGATCCAAGCGAT
WWP2	F	TGGAAGGCGGAAGTAGGA
	R	GTGAAGCTGGTGGAAGAGAAG
FBXW7	F	GTGAAGCTGGTGGAAGAGAAG
	R	TGCTCAGGCACGTCAGAAAAG
FASN	F	GAAGCTCGTGTTGACTTCTC
	R	AGAAGACCACAAAGTAGTCC
ACACA	F	AGTGGGTCACCCCATTGTT
	R	TTCTAACAGGAGCTGGAGCC
ACLY	F	GGTGCTCCGGATTTTGC
	R	ACATGGCTGCAGAGAGACCT
ACSS2	F	GGATTCCAGCTGCAGTCTTC
	R	CATGCCACCACAAGTCAATC
SCD1	F	GCAGCCGAGCTTTGTAAGAG
	R	GTTCTACACCTGGCTTTGGG
GCN5	F	GCAAGGCCAATGAAACCTGTA
	R	TCCAAGTGGGATACGTGGTCA
PCAF	F	AGGAAAACCTGTGGTTGAAGG
	R	CAGTCTTCGTTGAGATGGTGC
HDAC5	F	TCTTGTCGAAGTCAAAGGAGC
	R	GAGGGGAACTCTGGTCCAAAG
NOTCH1	F	GAGGCGTGGCAGACTATGC
	R	CTTGTACTCCGTCAGCGTGA
TCF7	F	CTGGCTTCTACTCCCTGACCT
	R	ACCAGAACCTAGCATCAAGGA

Supplementary Table 2

Sequences of the primers for qPCR

IL10	F	GGAGGAGGTGATGCCCCAAGCTGA
	R	TGCTCCACGGCCTTGCTCTTGT
CRLS1	F	AGCAGTCCAGTTAATCTTGGTG
	R	AGTCTTCCGGCCATAATGATAGT
CLN5	F	TAGGAGAAATTTCAGGCAAGCTG
	R	TATATGTGCGGTGAACAAACTGG
TGFB1	F	GGAAATTGAGGGCTTTCGCC
	R	CCGGTAGTGAACCCGTTGAT
